# A Stromal Immune Module Correlated with the Response to Neoadjuvant Chemotherapy, Prognosis and Lymphocyte Infiltration in *HER2*-Positive Breast Carcinoma Is Inversely Correlated with Hormonal Pathways

**DOI:** 10.1371/journal.pone.0167397

**Published:** 2016-12-22

**Authors:** Anne-Sophie Hamy, Hélène Bonsang-Kitzis, Marick Lae, Matahi Moarii, Benjamin Sadacca, Alice Pinheiro, Marion Galliot, Judith Abecassis, Cecile Laurent, Fabien Reyal

**Affiliations:** 1 Institut Curie, PSL Research University, Translational Research Department, INSERM, U932 Immunity and Cancer, Residual Tumor & Response to Treatment Laboratory (RT2Lab), Paris, France; 2 Department of Surgery, Institut Curie, Paris, France; 3 Department of Tumor Biology, Institut Curie, Paris, France; 4 Mines Paristech, PSL-Research University, CBIO-Centre for Computational Biology, Mines ParisTech, Fontainebleau, France; 5 U900, INSERM, Institut Curie, Paris, France; 6 Laboratoire de Mathématiques et Modélisation d’Evry, Université d’Évry Val d’Essonne, Evry, France; University of North Carolina at Chapel Hill School of Medicine, UNITED STATES

## Abstract

**Introduction:**

*HER2*-positive breast cancer (BC) is a heterogeneous group of aggressive breast cancers, the prognosis of which has greatly improved since the introduction of treatments targeting *HER2*. However, these tumors may display intrinsic or acquired resistance to treatment, and classifiers of *HER2*-positive tumors are required to improve the prediction of prognosis and to develop novel therapeutic interventions.

**Methods:**

We analyzed 2893 primary human breast cancer samples from 21 publicly available datasets and developed a six-metagene signature on a training set of 448 *HER2*-positive BC. We then used external public datasets to assess the ability of these metagenes to predict the response to chemotherapy (Ignatiadis dataset), and prognosis (METABRIC dataset).

**Results:**

We identified a six-metagene signature (138 genes) containing metagenes enriched in different gene ontologies. The gene clusters were named as follows: Immunity, Tumor suppressors/proliferation, Interferon, Signal transduction, Hormone/survival and Matrix clusters. In all datasets, the Immunity metagene was less strongly expressed in ER-positive than in ER-negative tumors, and was inversely correlated with the Hormonal/survival metagene. Within the signature, multivariate analyses showed that strong expression of the “Immunity” metagene was associated with higher pCR rates after NAC (OR = 3.71[1.28–11.91], *p* = 0.019) than weak expression, and with a better prognosis in *HER2*-positive/ER-negative breast cancers (HR = 0.58 [0.36–0.94], *p* = 0.026). Immunity metagene expression was associated with the presence of tumor-infiltrating lymphocytes (TILs).

**Conclusion:**

The identification of a predictive and prognostic immune module in *HER2*-positive BC confirms the need for clinical testing for immune checkpoint modulators and vaccines for this specific subtype. The inverse correlation between Immunity and hormone pathways opens research perspectives and deserves further investigation.

## Introduction

*HER2*-positive breast carcinomas (BCs) are defined by amplification and overexpression of the *HER2* tyrosine kinase receptor gene (17q12). The tumors of this subgroup have aggressive pathological features and a high rate of early distant metastatic events. They are routinely treated with a combination of docetaxel plus a monoclonal antibody targeting the *HER2* receptor (trastuzumab). Other drugs also appear to be of major interest and will probably be made available for routine treatment in the near future (lapatinib, pertuzumab and T-DM1).

*HER2*-positive BCs constitute a heterogeneous group of tumors differing in histological features, gene expression profiles, clinical behavior, overall prognosis, and response to conventional systemic cytotoxic therapy. Trastuzumab-based treatments have been used for the last decade and have substantially improved outcomes in patients with early or metastatic *HER2*-positive BC. However, some *HER2*-positive tumors display intrinsic or acquired resistance to trastuzumab. Robust classifiers are required, both to improve our understanding of the molecular basis of *HER2*-positive BC and to develop novel therapeutic interventions.

We developed a two-step biological network-driven gene selection process: 1) identification of the most variable genes displaying highly correlated patterns of expression, 2) direct connection of these genes within known biological networks. This method has been shown to construct molecular signatures efficiently [1–3]. We defined a *HER2*-positive molecular subtype classification and identified a stromal immune module gene expression profile strongly correlated with predicted response to chemotherapy, prognosis and lymphocytic infiltration. This classification provides considerable biological insight, and has potential for use in the development of therapeutic interventions, such as novel immunotherapies in particular.

## Material and methods

### Data normalization and quality control

#### Training, validation and Ignatiadis datasets

We collected 21 publicly available datasets (described in the S1 File) containing raw gene expression data for 2893 primary human breast cancer samples. The data were normalized by the robust multichip average (RMA) procedure from the EMA R package [4]. The datasets were split into training (HGU-133A Affymetrix* arrays, 12 datasets, *n* = 1921) and validation (HGU-133Plus2 Affymetrix* arrays (9 datasets, *n* = 972) sets. Batch effects were eliminated by the median centering of each probe-set across arrays and by an independent quantile normalization of all arrays for each dataset. We controlled for outliers with the Array Quality Metrics R package. We also collected two large datasets to validate our classification: The Ignatiadis dataset (Affymetrix data *n* = 996) [5] and the METABRIC dataset (Illumina data *n* = 1992) published by Curtis *et al*. [6].

### Determination and preprocessing of *HER2*-positive breast cancer samples

We identified the *HER2*-positive samples in the training and validation datasets, on the basis of transformed ERBB2 mRNA expression, as described by Gong *et al*. [7], and using the bimodal distribution of ERBB2 expression for the Ignatiadis and the METABRIC dataset.

### Gene selection process

Consensus clustering with the ConsensusClusterPlus R Package was applied to the training set with a ward inner, final linkage and Pearson distance, to determine the optimal number of robust gene clusters for the most variable genes (standard deviation>0.8). We investigated the enrichment of each gene cluster in particular types of genes, and categorized and labeled genes clusters according to the different gene ontologies. We then identified known biological networks, for each gene cluster separately, using String* database software version 9.1 (http://string-db.org/) [8]. We then applied a two-step selection process: 1) we selected strong biological networks by retaining only genes for which connection scores of at least 0.7 were obtained with String* database software, 2) within each biological network, we selected groups of genes with correlated expression patterns and a correlation coefficient of at least 0.5.

For each dataset (the training, validation, Ignatiadis and METABRIC sets), we applied a hierarchical clustering procedure with a ward inner, final linkage and Pearson distance to the *HER2*-positive gene expression (GE) profiles, using the selected genes to visualize the optimal number of stable *HER2*-positive subtypes.

### Metagene construction

We defined a metagene as an aggregate patterns of gene expression. Metagene expression was assessed by calculating the median normalized expression values of all probe sets in the respective gene clusters for each sample. The metagene value for each sample was then discretized on the basis of the median value, as “high” or “low”.

### Association between expression of the Immunity metagene and that of ESR1, PGR, and AR

All the analyses were performed on all four datasets (training, validation, Ignatiadis, METABRIC). The levels of expression of ESR1, PGR and AR were compared between “Immunity low” and “Immunity high” samples, by ANOVA. Levels of Immunity metagene expression were compared between samples positive and negative for ER, PR, and AR, by ANOVA. We also performed ANOVA for each gene of the Immunity metagene as a function of ER status.

### Analysis of the predicted response to NAC

We analyzed the predicted response to chemotherapy in the datasets published by Ignatiadis *et al*. [5]. Expression data were summarized by defining a metagene for each gene cluster. The clinical and pathological variables available for each dataset are described in S1 File. Qualitative variables were compared with logistic regression models.

### Prognostic analysis

Prognostic analysis was performed on the METABRIC set. Expression data were summarized by defining a metagene for each gene cluster. The clinical and pathological variables available for each dataset are described in S1 File. Survival analyses were performed for the whole population, and separately for ER-positive and ER-negative patients, by calculating Kaplan-Meier estimates of the survival function. The endpoint of these analyses was breast cancer-specific survival (BCSS). Survival curves were compared in log-rank tests. Hazard ratios were estimated with Cox’s proportional hazard model. Predictive and prognostic analyses were performed with the R survival package. Variables associated with pCR or BCSS with a *P*-value <0.10 in univariate analysis were included in the multivariate model. Variables with *P*-values <0.05 in multivariate analysis were considered statistically significant.

### Correlation with tumor-infiltrating lymphocyte levels

We downloaded the gene expression data from the REMAGUS 02 trial [9] and retrieved 27 samples for which paraffin-embedded tissue sections were available at our institution. All patients enrolled in this study gave their informed written consent. Histologic microbiopsy specimens were evaluated independently for the presence of a lymphocytic infiltrate (intratumoral TILs and stromal TILs by one BC pathologist (ML) and one breast physician (ASH) unaware of the gene expression classification. Percentages of TLs and StrL were compared, as a function of Immunity metagene status, in ANOVA. The correlations between Immunity metagene expression and the percentages of TLs and StrL were assessed by calculating Pearson’s correlation coefficient.

### Expression of the gene signature in human breast cancer cell lines

We downloaded the gene expression profiles of the human cancer cell lines from the Cancer Cell Line Encyclopedia (CCLE) [10] of Novartis/the Broad Institute and the Cancer Genome Project (CGP) [11] of the Sanger Institute. We normalized the data for all the cell lines from different tissues together.

### Statistical analysis

Data were processed and statistical analyses were carried out with R software version 3.1.2 [12] (www.cran.r-project.org).

## Results

### *HER2*-positive gene expression profiles identify six main gene clusters

**Fig 1:**
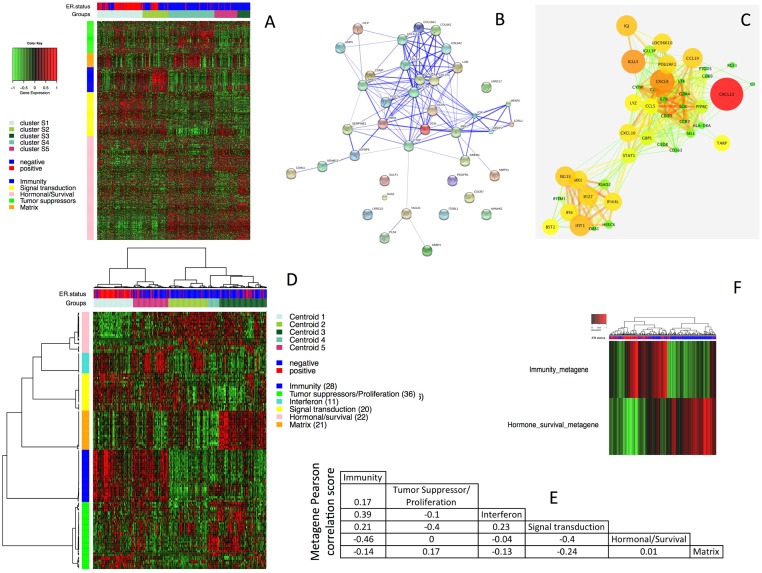
Gene selection process. **A** Heatmap showing the 616 most variable genes in the 448 *HER2*-positive samples (training set). **B** String database software confidence view of the Matrix genes cluster. Stronger associations between genes are represented by thicker lines. **C** Cytoscape View for the Immunity gene cluster. GE correlations between genes are indicated by edges (edge color varies from green to red and edge size increases with increasing correlation) and gene expression variance is represented by node color (node color varies from green to red and node size increases with increasing variance). **D** Heatmap showing the relative expression of 138 selected genes in 448 *HER2*-positive samples from the training set. **E** Table of Pearson’s correlation coefficient values for the correlations between the 6 metagenes. **F** Heatmap showing the anticorrelation between the Immunity and the Hormone/Survival metagene.

*HER2-*positive BC samples were selected from 21 publicly available datasets (n = 3,247 breast cancer samples) and separated into a training set and a validation set (S1 File and S1 Fig). In the training set, we applied a gene selection process based on biological networks (Fig 1A to 1C), to decrease the instability intrinsic to molecular classification methods (see S1 File), as previously described for triple-negative breast cancers (TNBCs) [3]. We selected a final set of 138 genes (S1 Table), composed of six gene clusters enriched in different gene ontologies: Immunity (*n* = 28), Interferon (*n* = 11), Signal transduction (*n* = 20), Hormonal/survival (*n* = 22), Tumor suppressors/Proliferation (*n* = 36), Matrix (*n* = 21) (Fig 1D). We defined a metagene for each of the six gene clusters identified in this way (S1 File). The Immunity and Interferon metagenes displayed similar patterns of expression. The Immunity and Hormonal/survival metagenes displayed the strongest inverse correlation for expression (coefficient of -0.46) (Fig 1E and 1F). The correlations between the 138 genes and the metagenes are described in more detail in S1 File. For validation, we applied hierarchical clustering methods to three additional independent *HER2*-positive datasets; a validation set (*n* = 194), the Ignatiadis dataset (*n* = 82) and the METABRIC dataset (*n* = 248) (S1 File and S2 Fig).

### The expression of the Immunity metagene is strongly associated with ER status, PR, and AR status

**Fig 2:**
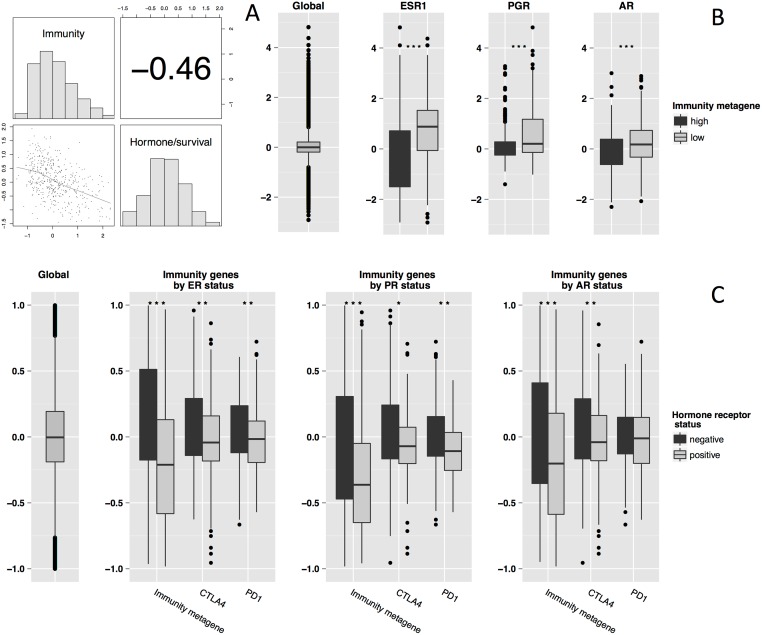
Association between Hormone genes expression and Immunity genes expression. **A** Correlation of Immunity metagene and Hormone/Survival metagene expression (training set). Pearson’s correlation coefficient is -0.46 (95% CI [-52.7–38.0], *p*<10^−16^). **B** Boxplots of global gene expression and ESR1, PGR and AR expression by Immunity metagene status, “low” versus “high” in the training set (A). P-values for ANOVA are *p* = 10^−16^, *p* = 10^−6^ and *p* = 0.0002, respectively. **C** Boxplots of Immunity metagene and immune gene (CTLA4 and PD1) expression levels by ER, PR and AR status in the training set (A). The *p* values for ANOVA were *p*<10^−16^, *p* = 0.002 and *p* = 0.008 for the Immunity metagene, CTLA4 and PD1 by ER status, respectively; *p* = 0.0001, *p* = 0.05 and *p* = 0.001 by PR status, respectively; and *p*<10^−6,^ *p* = 0.006 and *p* = 0.23 by AR status, respectively. The statistical significance (*p*-value) of the difference between gene expression values is indicated by black stars (*p*-value ≤ 0.05: *; *p*-value≤ 0.01: **; *p*-value≤ 0.001: ***).

Given the inverse correlation between Immunity metagene and the Hormonal/survival metagene expression (Fig 2A) and with the strong correlation of Hormonal/survival metagene expression with ESR1 expression (Pearson correlation coefficient = 0.77), we compared levels of ESR1, PGR and AR expression as a function of Immunity metagene status (Fig 2B). These three genes were consistently more strongly expressed in the “Immunity low” subgroup than in the “Immunity high” subgroup (*p*< 10^−16^, *p*< 10^−8^, *p* = 0.002 respectively). Similar results were obtained with the other three datasets, although less consistently for PR and AR (S1 File).

We then compared the levels of expression of our Immunity metagene with those of two other immune genes (CTLA4 and PD1; PDL1 was not available on the HGU133a Chip) as a function of ER, PR, and AR status. The Immunity metagene and CTLA4 were significantly more strongly expressed in the ER-negative, PR-negative, and AR-negative subgroups (Fig 2C). PD1 was significantly more strongly expressed in ER-negative and PR-negative tumors, but the difference in expression levels according to AR status was not significant for this gene. Similar findings were obtained when we compared each of the genes of the Immunity metagene separately as a function of ER status, and across the three other datasets. The results were less consistent for PR and AR (see S1 File). The proportions of tumors in the Immunity metagene “low” and “high” subgroups as a function of ER status differed significantly in three of the four datasets. ER-positive samples were more likely to be in the Immunity metagene “low” group, whereas ER-negative samples were more likely to be in the Immunity metagene “high” group (S1 File).

These findings suggest that there are strong inverse interactions between immune pathways that are captured by the Immunity metagene and ER, PR, and AR hormonal pathways in *HER2-*positive breast cancer tumors.

### Predictive value of the Immunity metagene in *HER2*-positive breast cancers

**Fig 3:**
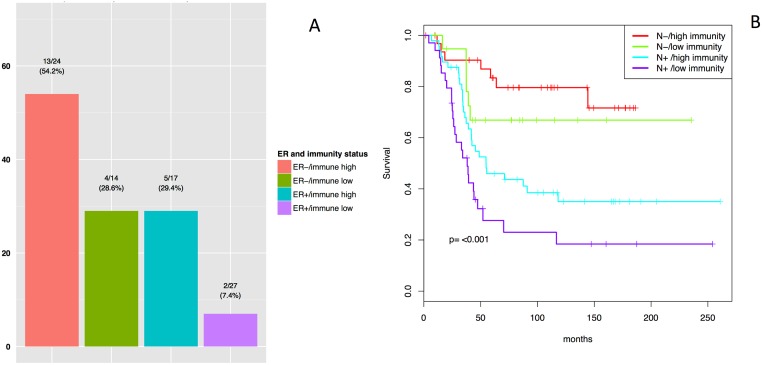
pCR and DSS outcomes in the Ignatiadis and the METABRIC dataset. **A:** pCR rates by ER and Immunity metagene status (low *versus* high in the Ignatiadis dataset). **B:** Kaplan-Meier plots. Disease-specific survival of the ER-negative population (*n* = 138) according to Immunity metagene expression (low/high) and nodal status in the METABRIC dataset.

We assessed the value of the six metagenes for predicting the response to neoadjuvant chemotherapy (NAC) on 82 *HER2*-positive samples from the Ignatiadis dataset. Univariate analysis identified four factors (ER status, tumor grade, and Immunity and Hormone/survival metagene expression) correlated with pathological complete response (pCR) (Table 1). In multivariate analysis, both ER status and the Immunity metagene were significantly associated with pCR (ER-positive: OR = 0.29 [0.09–0.82] versus ER-negative (reference class), *p* = 0.02; Immunity metagene “high” expression: OR = 3.71, 95% CI [1.28–11.91], *versus* “low” expression (reference class), *p* = 0.02) (Fig 3A). Analyses in the subset of patients that did not receive trastuzumab (*n* = 75) yielded similar results (S1 File).

**Table 1:** Association of clinical factors and gene cluster expression with pathological response rates after neoadjuvant chemotherapy in the Ignatiadis dataset, univariate and multivariate analysis.

		n	Univariate analysis			Multivariate analysis		
			OR	IC	*pval*	OR	IC	*pval*
Age	<50 y.o.	39	1					
	> = 50 y.o	43	1.1	[0.42–2.9]	0.84			
ER status	ER negative	38	1			1		
	ER positive	44	0.23	[0.08–0.63]	**0.006**	0.29	[0.09–0.82]	0.023
PR status	PR negative	78	1					
	PR positive	4	NA	NA*	0.99			
Tumoral size	T1 and T2	34	1					
	T3	21	0.34	[0.08–1.14]	0.096			
	T4	27	0.41	[0.12–1.23]	0.122			
Nodal status	N0	12	1					
	N1,N2 or N3	55	1.02	[0.26–5.1]	0.974			
Tumor grade	Grade I or II	24	1					
	Grade III	51	4.16	[1.22–19.26]	**0.037**			
Immunity metagene	low	41	1			1		
expression	high	41	4.57	[1.65–14.2]	**0.005**	3.71	[1.28–11.91]	**0.019**
Tumor suppressor/proliferation	low	41	1					
metagene	high	41	1.61	[0.62–4.3]	0.333			
Interferon metagene	low	41	1					
expression	high	41	0.49	[0.18–1.27]	0.149			
Signal transduction metagene	low	41	1					
expression	high	41	1.27	[0.49–3.33]	0.628			
Hormone/survival metagene	low	41	1					
expression	high	41	0.22	[0.07–0.61]	**0.005**			
Matrix metagene	low	41	1					
expression	high	41	1.27	[0.49–3.33]	0.628			

*: OR not available, no pCR in the PR-positive group

We compared the predictive value of the Immunity metagene with that of nine immune signatures or metagenes already validated as predictors of the response to chemotherapy for breast cancer, notably in *HER2-*positive BCs [13–18]. In multivariate analysis, the Immunity metagene and six of the other signatures or metagenes tested were identified as predictive of the response to chemotherapy. The smallest *p*-value obtained was that for our Immunity metagene (*p* = 0.019), OR = 3.71, 95% CI [1.28–11.91] (S2 Table).

We then investigated the reasons for which the Immunity metagene (28 genes) was predictive of pCR in *HER2-*positive BCs, whereas the Immunity2 metagene (47 genes) published by Bonsang *et al*. [3] was not in a TNBC population [3], despite the strong correlation between these two signatures in three independent datasets (correlation coefficients: 0.96; 0.94 and 0.96 in the training set, METABRIC and Ignatiadis dataset, respectively). We applied both signatures to the whole population for the Ignatiadis dataset, and analyzed pCR as a function of breast cancer subtype and Immunity metagene status. We found that pCR rates were significantly higher in the “Immunity high” subgroup in *HER2-*negative/ER-positive (16.7% *versus* 8.4%, OR = 2.17, *p* = 0.05), *HER2*-positive (43.6% *versus* 16.7%, OR = 3.84, *p* = 0.01), and TNBC breast cancers (37.3 *versus* 22.6%, OR = 2.08, *p* = 0.03) (S3A Fig). A similar pattern was observed for the Immunity2 metagene (*HER2-*negative-ER positive: 16.5% *versus* 8.1%, OR = 2.22, *p* = 0.05), *HER2*-positive (45.5% *versus* 18.7%, OR = 3.57, *p* = 0.01), and TNBC breast cancers (36.3 *versus* 24.6%, OR = 1.75, *p* = 0.08; S3B Fig), but the difference was not statistically significant (*p* = 0.08) in the TNBC subgroup. Interestingly, Immunity metagene status appeared to have a larger effect on pCR rates in the *HER2*-positive subgroup (OR = 3.84 and 3.57, respectively) than in the ER-positive (OR = 2.17 and 2.22, respectively) and TNBC (OR = 2.08 and 1.75, respectively) subgroups. The Immunity metagene therefore seems to be associated with the response to NAC in all breast cancer subtypes, with a marked effect in terms of both the strength and magnitude of the association in the *HER2-*positive subgroup.

### Prognostic value of the Immunity metagene in *HER2*-positive breast cancers

The prognostic value of the 138-gene *HER2*-positive signature was assessed with 248 *HER2*-positive samples from the METABRIC dataset. Univariate analysis identified five factors (menopausal status, tumor size, nodal status, Immunity and Signal transduction metagene expression) significantly correlated with a poor outcome (disease-specific survival) (Table 2).

**Table 2:** Survival analysis (disease-specific survival) in the METABRIC dataset (univariate and multivariate analysis); whole population and ER-negative population.

		Whole population (n = 248)							ER negative population (n = 138)						
		n	Univariate analysis			Multivariate analysis			n	Univariate analysis			Multivariate analysis		
			HR	IC	*pval*	HR	IC	*pval*		HR	IC	*pval*	HR	IC	*pval*
Age at diagnosis	< = 45 y.o.	52	1		-				25	1					
	45–55	59	0.67	[0.4–1.14]	0.142				19	0.65	[0.36–1.18]	0.153			
	>55	130	0.66	[0.43–1.04]	**0.071**				23	0.62	[0.35–1.1]	0.103			
Menopausal status	Pre	74	1		-				32	1					
	Post	167	0.68	[0.46–1]	**0.051**				33	0.67	[0.41–1.09]	0.11			
Tumoral size	< 20 mm	68	1		-				15	1					
	> = 20 mm	173	1.87	[1.18–2.96]	**0.008**				52	1.51	[0.85–2.69]	0.159			
Tumor grade	I	3	1		-				10	1	-	-			
	II	53	1.66	[0.22–12.19]	0.621				55	0.942	[0.48–1.85]	0.863			
	III	178	1.81	[0.25–13.05]	0.554				10	NA	NA	NA			
ER status	negative	135	1		-										
	positive	108	0.74	[0.51–1.07]	0.108										
PR status	negative	193	1		-				65	1					
	positive	50	0.84	[0.53–1.34]	0.46				2	2.3	[0.56–9.49]	0.25			
Nodal status	N-	105	1		-	1			13	1			1		
	N+	138	3.26	[2.13–5.01]	**<0.001**	3.29	[2.14–5.06]	**<0.001**	54	3.55	[1.93–6.51]	**<0.001**	3.57	[1.94–6.55]	**<0.001**
NPI	GP	38	1		-				6	1					
	IP	155	1.26	[0.71–2.25]	0.433				35	1.01	[0.42–2.4]	0.988			
	PP	50	3.32	[1.78–6.19]	**<0.001**				26	2.81	[1.15–6.84]	**0.023**			
Metagene expression															
Immunity	low	122	1		-	1			54	1					
	high	121	0.71	[0.49–1.03]	**0.073**	0.70	[0.48–1.01]	**0.054**	81	0.58	[0.36–0.94]	**0.028**	0.58	[0.36–0.94]	**0.026**
TS /proliferation	low	121	1		-				50	1					
	high	122	1.04	[0.72–1.51]	0.828				85	0.84	[0.51–1.38]	0.491			
Interferon	low	122	1		-				78	1					
	high	121	1.23	[0.85–1.78]	0.278				57	1.28	[0.79–2.07]	0.316			
Signal transduction	low	121	1		-				72	1					
	high	122	1.48	[1.02–2.14]	**0.04**				63	1.34	[0.83–2.17]	0.232			
Hormone/survival	low	122	1		-				114	1					
	high	121	0.94	[0.65–1.36]	0.751				21	1.35	[0.72–2.52]	0.351			
Matrix	low	121	1		-				69	1					
	high	122	1.05	[0.73–1.52]	0.785				66	1.03	[0.64–1.67]	0.889			

Abbreviations: GP: good prognosis, IP: intermediate prognosis, PP: poor prognosis; TS: tumor suppressor

In multivariate analysis, nodal status (node-negative *versus* node-positive) was significantly associated with a poor outcome (HR = 3.29 [2.14–5.06], *p*<0.001), and there was a trend towards association between high levels of Immunity metagene expression and better disease-free survival (DFS; HR = 0.70 [0.48–1.01], *p* = 0.054). In the ER-negative population, the Immunity metagene was found to be of significant prognostic value in multivariate analysis (*n* = 138) (HR = 0.58 [0.36–0.94], *p* = 0.026; Fig 3B), but was not associated with DFS in the ER-positive population (*n* = 110) (*p* = 0.43). We compared the prognostic value of the Immunity metagene with that of nine previously published immune signatures or metagenes known to predict survival in several breast cancer subtypes [14,17–22]. None of the signatures or metagenes described above was significantly associated with prognosis (S2F Table).

### The Immunity metagene is correlated with tumor-infiltrating lymphocytes (TILs) in *HER2*-positive breast cancer

**Fig 4:**
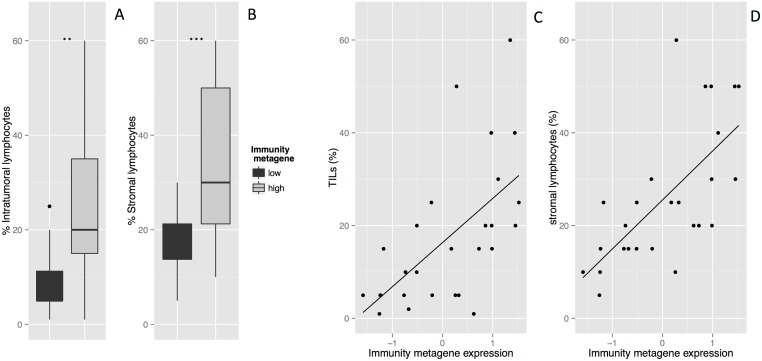
Association between tumor-infiltrating lymphocyte levels and Immunity metagene expression in the REMAGUS dataset. **A:** Percentage of intratumoral TILs according to Immunity metagene status (low *versus* high). **B** Percentage of stromal TILs according to Immunity metagene status (low *versus* high). **C**: Correlation between metagene expression and the percentages of intratumoral TILs. **D**: Correlation between metagene expression and the percentage of stromal TILs.

**Fig 5:**
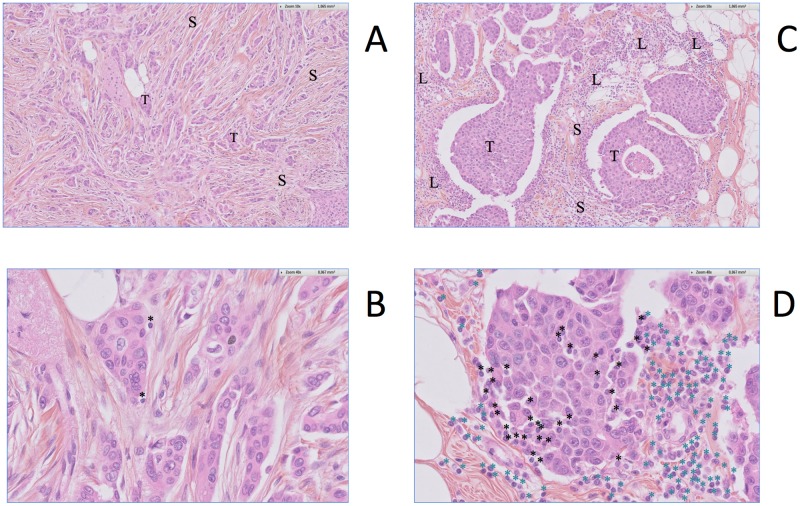
Lymphocytic infiltration in breast tumors. **A** and **B:** Tumor specimen with weak lymphocytic infiltration (A: zoom x10 B: zoom x 40). Abbreviations: S = stroma, T = tumor, L = lymphocytes. Intratumoral TILs are indicated by a black star. **C** and **D**: Tumor specimen with prominent lymphocytic infiltration. (C: zoom x10 D: zoom x 40). Abbreviations: S = stroma, T = tumor, L = lymphocytes. Intratumoral TILs are indicated by a black star; stromal TILs are indicated by a blue star.

We then investigated the correlation between Immunity metagene expression and lymphocyte infiltration. We analyzed an independent set of *HER2*-positive tumors for which both histology and gene expression data were available (*n* = 27). Intratumoral TILs (TLs) and stromal TILs (StrL) were evaluated separately. Intratumoral TIL percentages were significantly higher in patients with strong Immunity metagene expression than in those with weak Immunity metagene expression (24% and 9%, respectively, *p* = 0.001) (Fig 4A). The same pattern was observed for the percentage of stromal TILs (36% versus 16.6%, *p* = 0.009) (Fig 4B). The coefficients of correlation between Immunity metagene expression level on the one hand and the percentage of intratumoral TILs (Fig 4C) or stromal TILs (Fig 4D) on the other hand were high (*r* = 0.60, *p*<0.001 and *r* = 0.69, *p*<0.00001 respectively). Lymphocyte infiltration is shown for two specimens, one with weak (Fig 5A and 5B), and the other with strong lymphocyte infiltration (Fig 5C and 5D). The Immunity metagene was therefore strongly correlated with the amount of lymphocyte infiltration in both the stromal compartment and the tumor bed.

### The Immunity metagene corresponds to the B-cell, T-cell and CD8 cell pathways

The Immunity metagene was strongly correlated with several published immune signatures (S4 Fig and S1 File), suggesting the use of similar immune pathways (see S1 File). We analyzed the correlation between expression of the Immunity and Interferon metagenes and expression of the metagenes defined by Gatza *et al*. [23] (IFN-alpha, IFN-gamma, STAT3, TGF-beta, TNF-alpha) and Palmer *et al*. [24] (LB, LT, CD8, GRANS, LYMPHS). This analysis was performed on the METABRIC dataset. The Immunity metagene was highly correlated with the B-cell, T-cell and CD8 cell metagenes (Pearson correlation coefficients: 0.89, 0.86, and 0.90, respectively; S5 Fig). We also assessed the correlations between the expression of PD1, PDL1, CTLA4, and that of their respective metagenes. The PD1 and CTLA-4 metagenes were constructed from the genes most strongly correlated with the PD1 and CTLA-4 genes, respectively (Pearson’s correlation coefficient > 0.8). The PDL1 metagene was defined by Sabatier *et al*. [25]. Pearson’s correlation coefficients for the relationships between the Immunity metagene and each individual gene were strong for PD1 and CTLA-4 (Pearson’s correlation coefficient: 0.75 and 0.84, respectively), and weaker for PDL1 (0.36), but the expression of all three metagenes was strongly correlated with that of the Immunity metagene (Pearson’s correlation coefficient: PD1: 0.89, PDL1: 0.95, CTLA-4: 0.93), opening up new possibilities for therapeutic intervention.

### The Immunity metagene is probably expressed by stromal cells

In breast cancer cell lines (CCLE and CGP datasets), the Immunity metagene displayed very low levels of expression, similar to those of the CD8 metagene (S6A and S6B Fig), consistent with expression only in the tumor stromal compartment. This pattern was observed for all cell lines and breast cancer cell lines tested. The Interferon module genes had higher median expression levels and a broader range of expression than those of the Immunity metagene in breast cancer cell lines, consistent with their expression by tumor cells. We also explored the contributions of stromal and cancer cells to the expression of the Immunity and Interferon metagenes in detail, by comparing our gene lists with the “stromal contribution to global gene expression evaluated in PDX RNAseq data”, as defined by Isella *et al*. [26]. The stromal fraction of the Immunity metagene was high, although lower than those of the Matrix and the Tumor suppressor/proliferation metagenes. The Interferon metagene had a low stromal fraction, like the Hormone/survival and Signal transduction metagenes (S6C Fig). Although these data relate to the colon cancer PDX model, they provide support for the stromal expression of the Immunity metagene.

## Discussion

By analyzing the gene expression profiles of 448 *HER2*-positive breast cancers, we identified a six-metagene signature (138 genes) in which each of the various metagenes was enriched in a different gene ontology. Within these metagenes, we identified an immune stromal module inversely correlated with the ER and hormonal pathways and strongly associated with the predicted response to chemotherapy, prognosis, and tumor lymphocyte infiltration. We report here one of the first immune signatures identified as both predictive and prognostic, reflecting histological immune infiltration in *HER2*-positive breast cancers. We also provide a relevant analysis by HR status.

We previously developed a strategy for defining gene expression signatures based on the analysis of biological networks for the most variable genes [3]. Since the early 2000s, a molecular classification of breast cancers has emerged that is continually being refined. Several authors have proposed TNBC subclassifications [3,27,28] but, to our knowledge, only one classifier has been published, but was not subsequently validated in *HER2*-positive BC [18]. The various metagenes in our signature were enriched in different gene ontologies: two clusters were enriched in immunity genes, one in signal transduction genes, one in hormonal/survival genes, one in tumor suppressor/proliferation genes and one in matrix genes. Unlike several other teams [29–31], we did not identify a subgroup to tumors overexpressing androgen receptor pathways in *HER2*-positive BCs by our biology-driven approach. The expression of the Immunity and Hormone/survival metagenes accurately predicted the response to NAC, but the expression of the Hormone/survival metagene had no significant effect in multivariate analysis, because the information it provided largely overlapped with ER status. Moreover, only the Immunity metagene was found to be of significant prognostic value.

Several authors have previously identified immunity patterns in *HER2*-positive BC. The Immunity module identified in our study had many biological connections with other predictive or prognostic immune signatures published for *HER2*-positive breast cancers [13–21], but it outperformed previous classifiers. This module includes genes encoding chemokines for T cells (CXCL10, CXCL9, CCL5), B cells (CXCL13), both B and T cells (CCL19) or other immune cells (CXCL13, CCL5); chemokine receptors (CCR7); cytokines (LTB); adhesion molecule-associated genes (SELL), and genes encoding proteins involved antigen processing and presentation (HLA-DRA), B-lymphocyte cell surface molecules (PTPRC, HLA-DRA), complement pathway proteins (C1QB), and proteins involved in CTL-mediated immune responses to target cells (CD3D), dendritic cell regulation of Th1 and Th2 development (CD2, IL7R), granzyme-mediated apoptosis (GZMA), IL12-mediated signaling events (CD3D, HLA-DRA, GZMA, LCK), the IL2 signaling pathway (LCK), T-cell surface molecules (PTPRC, CD3D, CD2), and molecules of the T-cell receptor signaling pathway (PTPRC, CD3D, HLA-DRA, LCK). It was also strongly correlated with the B-cell, T-cell and CD8 cell pathways.

There was a marked significant inverse association between ESR1 expression and that of the Immunity metagene. Similar inverse associations were found between PGR, AR and immunity, but these associations were weaker and less consistent. There is growing evidence for sex-based differences in the innate and adaptive immune responses underlying susceptibility to infectious diseases and the prevalence of autoimmune diseases. A higher proportion of men than of women display infectious diseases and their severity is also greater in men than in women [32]. By contrast, many autoimmune diseases predominantly affect women [33]. There are also difference between men in terms of humoral and cellular responses to infection and vaccination, with women often displaying higher response rates and mounting stronger humoral responses [34]. Estrogen receptors are expressed in most of the cells of the innate and adaptive immune system, including T cells, B cells, neutrophils, macrophages, dendritic cells (DC), and natural killer (NK) cells [35]. The effects of major sex steroid hormones were reviewed by Giefing-Kröll [36]. Estradiol and testosterone have opposite effects on the cells of the adaptive and innate immune systems, with estradiol having mostly enhancing and testosterone mostly suppressive effects. Estrogens affect the expression of some chemokine receptors (CCR1 and CCR5) by T cells [37]. They also affect B-cell development [38], decrease the cytotoxicity of NK cells [39] and regulate DC development [40]. TReg-cell frequencies within the CD4^+^ population change considerably during the ovarian cycle, with potential effects on immunoregulation [41]. Unlike the differences between the sexes in terms of infection and auto-immunity, the relationships between tumor immunology, sex and steroid hormones have remained largely unexplored. In two phase III trials, immunotherapy had a significant beneficial effect on survival only in male patients [42,43]. However, it remains unclear whether there is a true “sex” effect on the efficacy of immunotherapy or whether these findings are purely incidental.

The interaction between the ER, immunity and *HER2* pathways is complex. There is increasing evidence to suggest that interactions between *HER2* and hormone-receptor pathways play an important role in disease progression and that there is extensive, complex, bidirectional, crosstalk between the *HER2* and ER pathways [44]. Immune signatures have been reported to have a predictive or prognostic role mostly in ER-negative breast cancers [45–48]. In *HER2-*positive breast cancer subtypes, Rody found that an immune T-cell metagene was of predictive value in both ER-positive and ER-negative *HER2*-positive BC [49]. The prognostic value of HDDP was demonstrated in both subgroups (11), but its value for predicting the response to NAC was not evaluated as a function of ER status. Conversely, the IRSN-23 [15] was not predictive in the ER-positive subpopulation. However, few authors determined the predictive [18] or prognostic value of their metagene or signature as a function of ER status within *HER2*-positive breast cancers [5,13,14,19–21]. The inverse association observed between ESR1 expression and immunity genes may be an important piece of the puzzle, and merits further investigation.

Consistent with previous reports [13,15,16], we found that the Immunity metagene was predictive of the response to NAC in *HER2*-positive BC. However, despite the similar gene module identification methods used and the strong correlation between the Immunity metagene and the Immunity2 metagene previously described by our team for TNBC [3], the Immunity metagene was predictive of the response to chemotherapy in *HER2-*positive BC, whereas the Immunity2 metagene was not predictive of the response to chemotherapy in TNBC. This finding was reported in the princeps report by Ignatiadis, in which high immune module scores were strongly and independently associated with a higher probability of pCR probability in *HER2*-positive tumors, whereas this association, although still significant, was weaker in TNBC [5]. ER-positive tumors have long been described as chemoresistant, with low pCR rates after NAC. Taking Immunity metagene expression into account, pCR rates ranged from 7.4 to 29.4%, with the highest rates close to those of ER-negative tumors.

The Immunity metagene was also prognostic in *HER2*-positive ER-negative breast cancer. The impact of immunity on prognosis has been reported before [21](Alexe et al., 2007)[21]^14^[18,20,21]. Together with our work, these findings suggest that immunity gene expression is highly predictive and of prognostic value in *HER2-*positive breast cancer. Nevertheless, the *HER2*-positive patients of the METABRIC dataset did not receive targeted anti-*HER2* therapies, and our results would probably be influenced by adjuvant trastuzumab treatment.

We also demonstrated a correlation between Immunity metagene expression and stromal and intratumoral lymphocyte infiltration. The significance of TILs has recently become apparent, with advances in tumor immunology and the availability of cancer immunotherapies. TIL levels are strongly correlated with breast cancer subtype, and are higher in *HER2*-positive BCs than in ER-positive BCs, but lower than in TNBCs [50]. TIL levels are consistently higher in ER-negative tumors than in ER-positive tumors [51]. This was also found to be the case when the analysis was limited to *HER2*-positive BC only [52], [50]. The value of TIL levels for predicting pCR after NAC is less clear in *HER2*-positive BC than in TNBC. Stromal TILs and the lymphocyte-predominant breast cancer phenotype (LPBC) were strongly associated with treatment response in the GeparSixto trial [13]. However, this effect was found to be nonlinear in the NeoALTTO trial, and the optimal cutoff value remains unclear [52]. Two large studies in the adjuvant setting gave conflicting results. A positive association between higher levels of TILs and greater benefit from trastuzumab in *HER2*-positive disease was found in a retrospective analysis of the FinHER trial [50], whereas the opposite result was reported in the ALLIANCE N9831 study [53]. No difference in DFS between chemotherapy and chemotherapy plus trastuzumab was found in LPBC, whereas benefits of trastuzumab in addition to chemotherapy were observed only in non-LPBC. Thus, the prognostic impact of TILs on survival remains a matter of debate in *HER2-*positive BC. A few authors have reported a correlation between TIL and stromal lymphocyte levels and gene expression in *HER2*-positive breast cancers [13,15,21]. If this correlation is further validated, TIL levels could be used as a surrogate marker for the Immunity metagene, as TIL assessment is carried out in routine practice and is currently undergoing standardization [54].

## Conclusion

Our work opens up a number of exciting therapeutic perspectives in *HER2-*positive breast cancers. Due to the high immunogenicity of *HER2*-positive breast cancers and the considerable predictive and prognostic impact of immunity in this subtype, immunotherapies may soon become part of the therapeutic arsenal for such cancers. Preclinical models have suggested that there is synergy between anti-*HER2* monoclonal antibody and anti-PD-1 [55] or anti-CTLA4 antibodies [56]. The PANACEA phase Ib/II trial is currently investigating the use of pembrolizumab (KEYTRUDA^®^) in combination with trastuzumab, to determine whether the addition of an anti-PD-1 treatment can overcome trastuzumab resistance in patients with *HER2*-positive breast cancer whose cancer spread whilst they were on trastuzumab. Future challenges in the field of immunity and *HER2*-positive breast cancers include:

1. The public accessibility of large sets of gene expression data for tumors from patients treated with *HER2-*targeting treatments. As treatments are constantly changing for this breast cancer subtype, it is important for expression data to be shared promptly, to facilitate comprehensive research and the identification of predictive and prognostic markers in patients treated with cutting edge care.
2. Improvements in our understanding of hormone and immunity pathways in *HER2-*positive breast cancers. In particular, it would be very useful to determine whether a subset of patients with *HER2*-positive ER-positive cancers could be effectively treated by a combination of endocrine therapy/immune checkpoint blockade/ targeted therapy, without the need for chemotherapy.
3. Drug positioning strategies in HER2-positive BC, because, by contrast to other breast cancer subtypes, the *HER2*-targeting drug pipeline contains many candidates despite the comparative rarity of this particular disease.
4. The selection criteria for the candidates most likely to benefit from immune checkpoint blockade is a key point. The use of PD-L1 as a surrogate marker of anti-PD-1 efficacy remains controversial, even in cancers for which immunotherapy treatments have proved effective, and few data are available for breast cancer. The standardization and demonstrations of the reproducibility of published immune signatures would be useful, as would improvements in our understanding of the prognostic value of TILs in *HER2-*positive breast cancers. Moreover, it remains to be determined whether and how the immunogenic power of tumors with low expression of immunity genes could be enhanced.

Once these challenges have been overcome, given the outstanding results of immunotherapy for other cancers (e.g. melanoma, lung cancer) and the expected efficacy of such treatment for *HER2*-positive disease, such therapies could revolutionize the course of *HER2-*positive breast cancer in the near future.

## Supporting Information

S1 Fig Methodology flow chart. (PDF)

S2 Fig Heatmaps of the selected genes in the *HER2*-positive datasets. Training set (upper left); validation set (upper right), Ignatiadis (lower left), METABRIC (lower right).
(JPG)

S3 Fig pCR rates by breast cancer subtype and Immunity metagene. A: pCR rates by breast cancer subtype by Immunity metagene status (low *versus* high). B: pCR rates by breast cancer subtype by Immunity2 metagene status (low *versus* high) as previously published by Bonsang et al [3].
(PDF)

S4 Fig Heatmaps of the gene expression profiles of published immune signatures and connections between all immune genes. Fig A. Heatmap of the gene expression profiles of the nine immune predictive signatures or metagenes previously published, applied to the Ignatiadis dataset. The samples were ordered according to our classification of Low/High ‘Immunity’ metagene expression. B: Heatmap of the gene expression profiles of the immune prognostic signatures or metagenes previously published, applied to the METABRIC dataset. The samples were ordered according to our classification of Low/High ‘Immunity’ metagene expression. C: String Software connections between genes of our Immunity metagenes and the genes of previously published predictive or prognostic immune signatures or metagenes. Stronger associations between genes are represented by thicker lines. Associations between genes with a coefficient < 0.9 are shown in green. Associations between genes with a coefficient ≥ 0.9 are shown in red. Associations between genes with a coefficient between 0.4 to 0.7 are not shown.
(PDF)

S5 Fig Distribution histograms for our Immune metagenes and immune pathways. Distribution histograms for our Immune metagenes (Immunity and Interferon) and the immune pathway metagenes published by Gatza *et al*. (Interferon alpha, Interferon gamma, STAT3, TGF beta, TNF alpha) and Palmer *et al*. (B Cell, T Cell, CD8 T Cells, Granulocytes, Lymphocytes), Pearson correlation coefficient values and pairwise scatter plots.
(PDF)

S6 Fig Gene expression for the Immune metagenes and pathway, in cell lines and xenografts. A. Boxplots of gene expression for the Immune metagenes, the immune pathway metagenes (published by Gatza *et al*. and Palmer *et al*.) and the PD1, PDL1, CTLA4 metagenes in breast cancer cell lines from the CCLE (A) and the CGP (B). C: Boxplots of the stromal contribution to global gene expression evaluated with PDX RNAseq data (Isella *et al*.), for each of the gene clusters for our signature.
(PDF)

S1 File Supplementary methods and results. (PDF)

S1 Table 138-gene signature. (XLS)

S2 Table Association of published immune signatures or metagenes with response to chemotherapy and prognosis. Response to chemotherapy is assessed in the Ignatiadis dataset (univariate and multivariate analysis) (S2A to S2E Table). The association of published immune signatures or metagenes with prognosis is assessed in the METABRIC dataset (univariate analysis) (S2F Table).
(XLS)

## Abbreviations

BC: breast cancer
BCSS: breast cancer-specific survival
BC_CL: breast cancer cell lines
CCLE: Cancer Cell Line Encyclopedia
CGP: Cancer Genome Project
ER: estrogen receptor
GE: gene expression
*HER2*-positive BC: *HER2*-positive breast cancer
*HER2*: human epidermal growth factor receptor 2
PR: progesterone receptor
pCR: pathological complete response
RMA: robust multichip average
TILs: tumor-infiltrating lymphocytes
