# Supplementary material for: A Stromal Immune Module Correlated with the Response to Neoadjuvant Chemotherapy, Prognosis and Lymphocyte Infiltration in HER2-Positive Breast Carcinoma Is Inversely Correlated with Hormonal Pathways
Source: PLoS One. 2016 Dec 22;11(12):e0167397. doi: 10.1371/journal.pone.0167397 (PMC5178998; doi:10.1371/journal.pone.0167397)
Supplement: S1 Fig — (PDF) [file pone.0167397.s001.pdf]

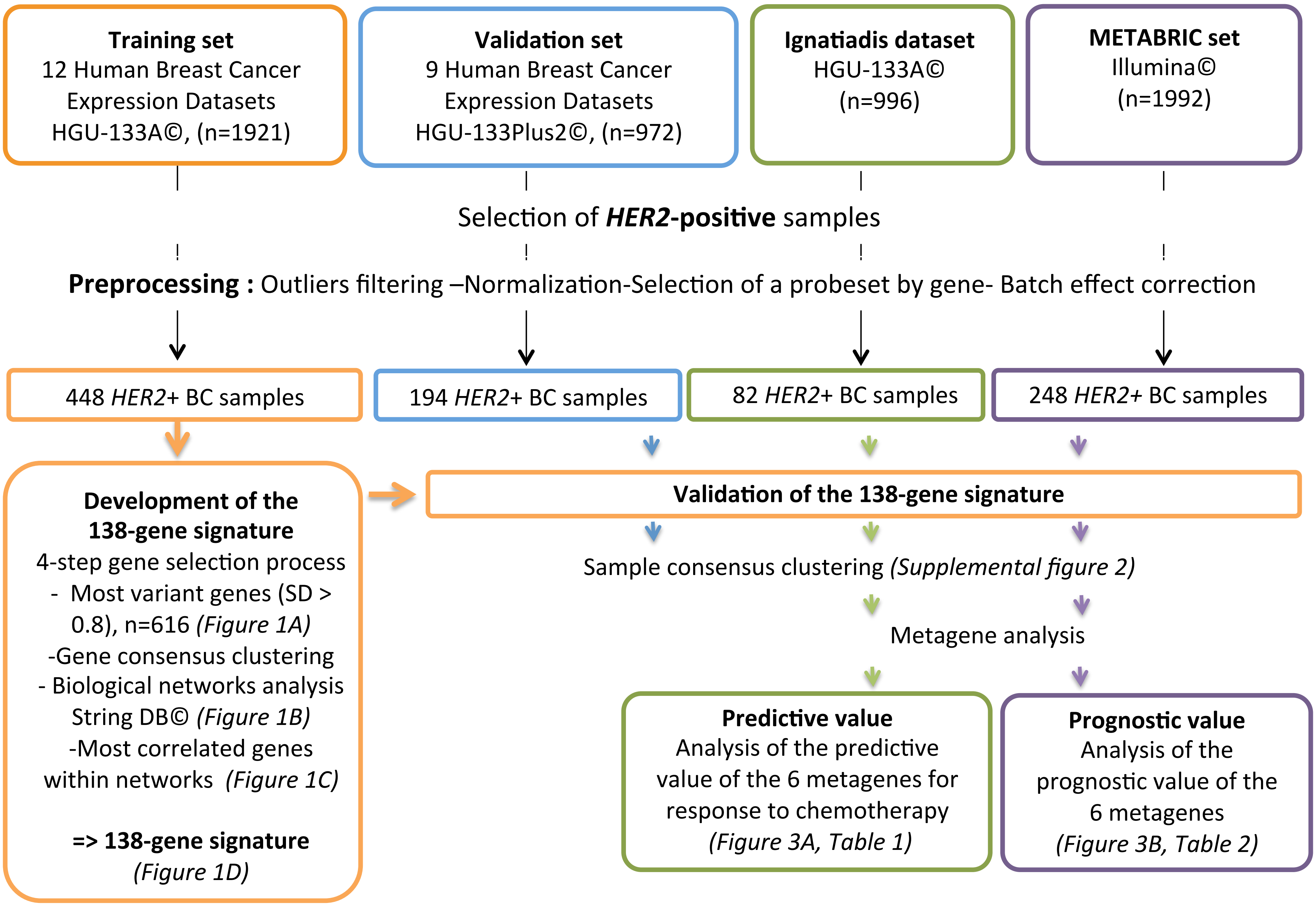
Training set
12 Human Breast Cancer Expression Datasets
HGU-133A©, (n=1921)
Validation set
9 Human Breast Cancer Expression Datasets
HGU-133Plus2©, (n=972)
Ignatiadis dataset
HGU-133A©
(n=996)
METABRIC set
Illumina©
(n=1992)
Selection of HER2-positive samples
Preprocessing : Outliers filtering –Normalization-Selection of a probeset by gene- Batch effect correction
448 HER2+ BC samples
194 HER2+ BC samples
82 HER2+ BC samples
248 HER2+ BC samples
Development of the 138-gene signature
4-step gene selection process
- Most variant genes (SD > 0.8), n=616 (Figure 1A)
-Gene consensus clustering
- Biological networks analysis String DB© (Figure 1B)
-Most correlated genes within networks (Figure 1C)
=> 138-gene signature (Figure 1D)
Validation of the 138-gene signature
Sample consensus clustering (Supplemental figure 2)
Metagene analysis
Predictive value
Analysis of the predictive value of the 6 metagenes for response to chemotherapy
(Figure 3A, Table 1)
Prognostic value
Analysis of the prognostic value of the 6 metagenes
(Figure 3B, Table 2)
