# Supplementary figures and images for: A Stromal Immune Module Correlated with the Response to Neoadjuvant Chemotherapy, Prognosis and Lymphocyte Infiltration in HER2-Positive Breast Carcinoma Is Inversely Correlated with Hormonal Pathways
Source: PLoS One. 2016 Dec 22;11(12):e0167397. doi: 10.1371/journal.pone.0167397 (PMC5178998; doi:10.1371/journal.pone.0167397)

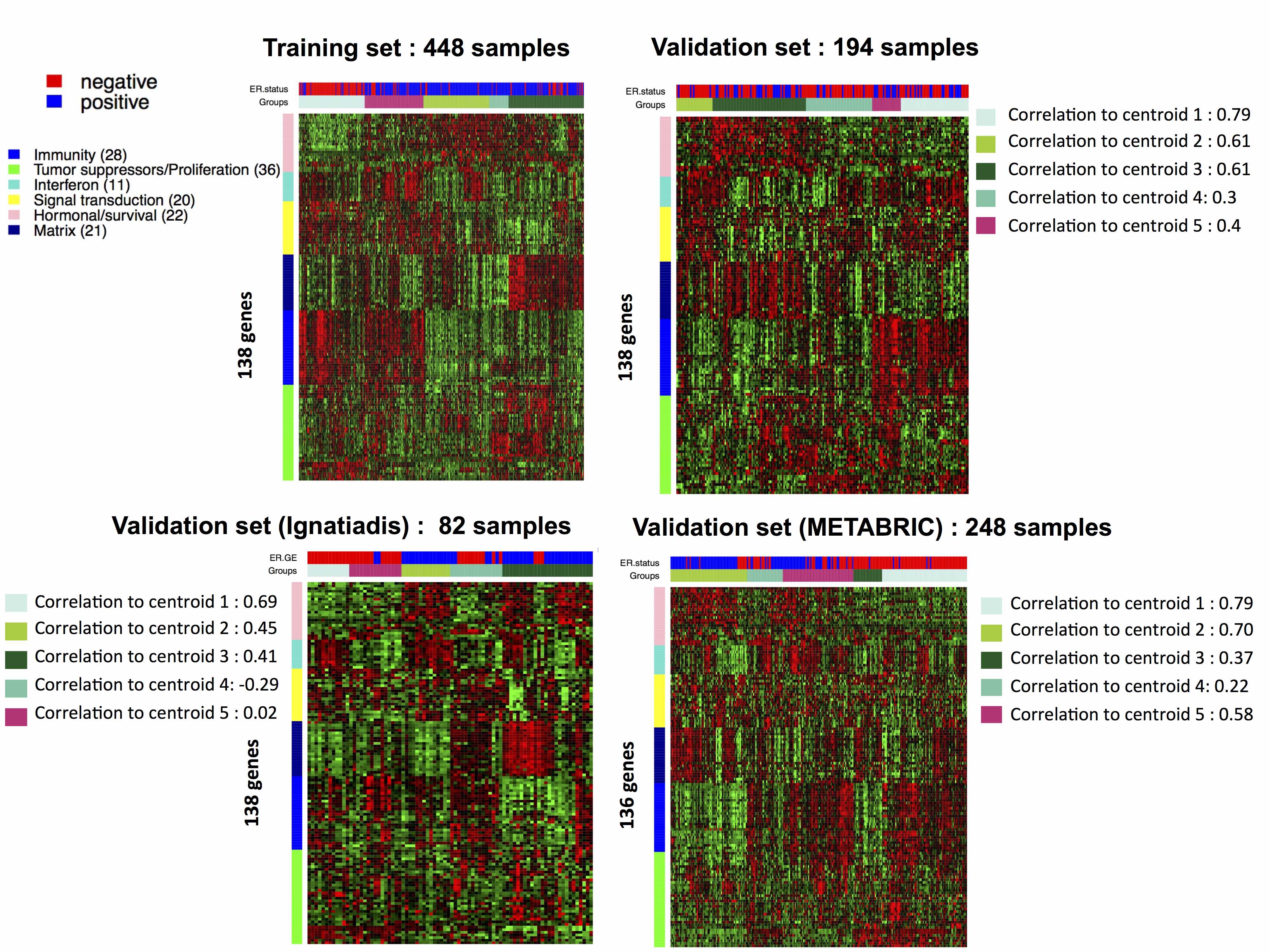

Supplement: S2 Fig — Training set (upper left); validation set (upper right), Ignatiadis (lower left), METABRIC (lower right). (JPG) [file pone.0167397.s002.jpg]

A

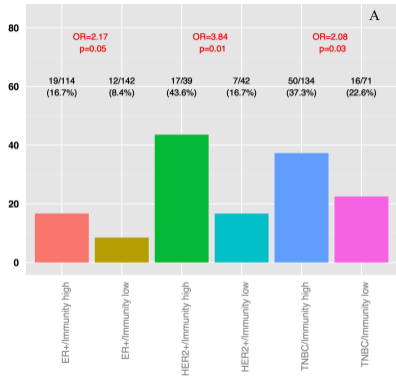

B

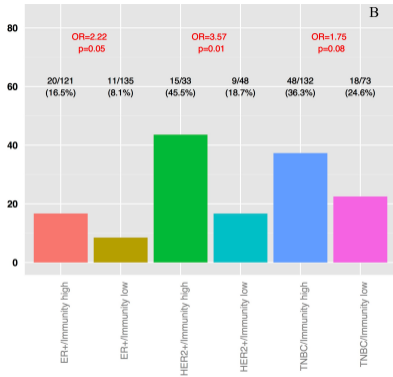

Supplement: S3 Fig — A: pCR rates by breast cancer subtype by Immunity metagene status (low versus high). B: pCR rates by breast cancer subtype by Immunity2 metagene status (low versus high) as previously published by Bonsang et al [3]. (PDF) [file pone.0167397.s003.pdf]

A

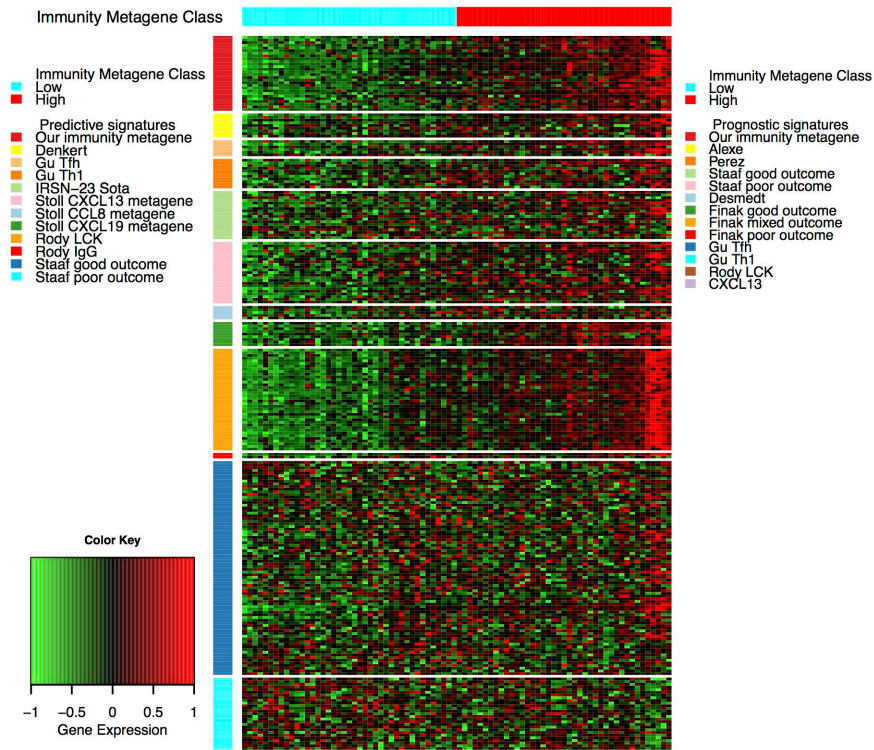

B

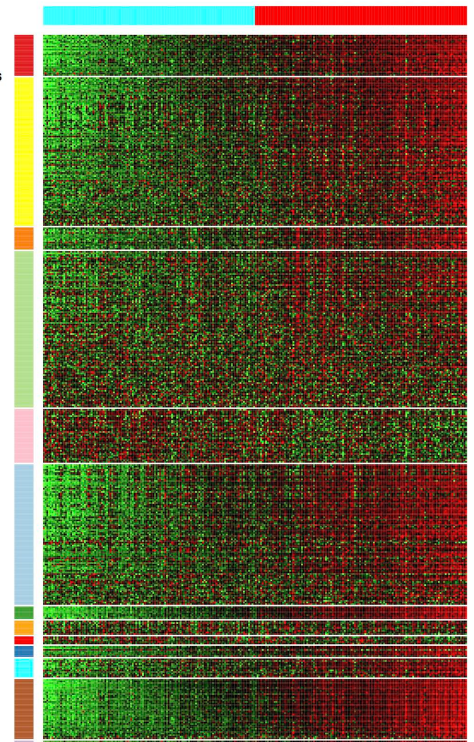

C

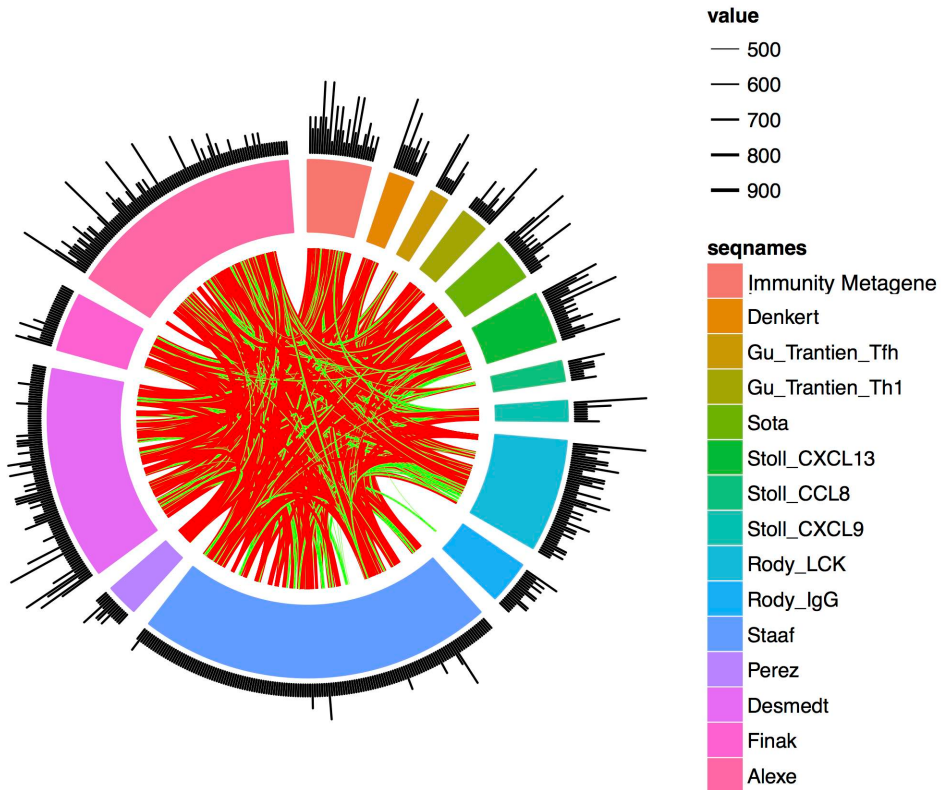

Supplement: S4 Fig — Fig A. Heatmap of the gene expression profiles of the nine immune predictive signatures or metagenes previously published, applied to the Ignatiadis dataset. The samples were ordered according to our classification of Low/High ‘Immunity’ metagene expression. B: Heatmap of the gene expression profiles of the immune prognostic signatures or metagenes previously published, applied to the METABRIC dataset. The samples were ordered according to our classification of Low/High ‘Immunity’ metagene expression. C: String Software connections between genes of our Immunity metagenes and the genes of previously published predictive or prognostic immune signatures or metagenes. Stronger associations between genes are represented by thicker lines. Associations between genes with a coefficient < 0.9 are shown in green. Associations between genes with a coefficient ≥ 0.9 are shown in red. Associations between genes with a coefficient between 0.4 to 0.7 are not shown. (PDF) [file pone.0167397.s004.pdf]

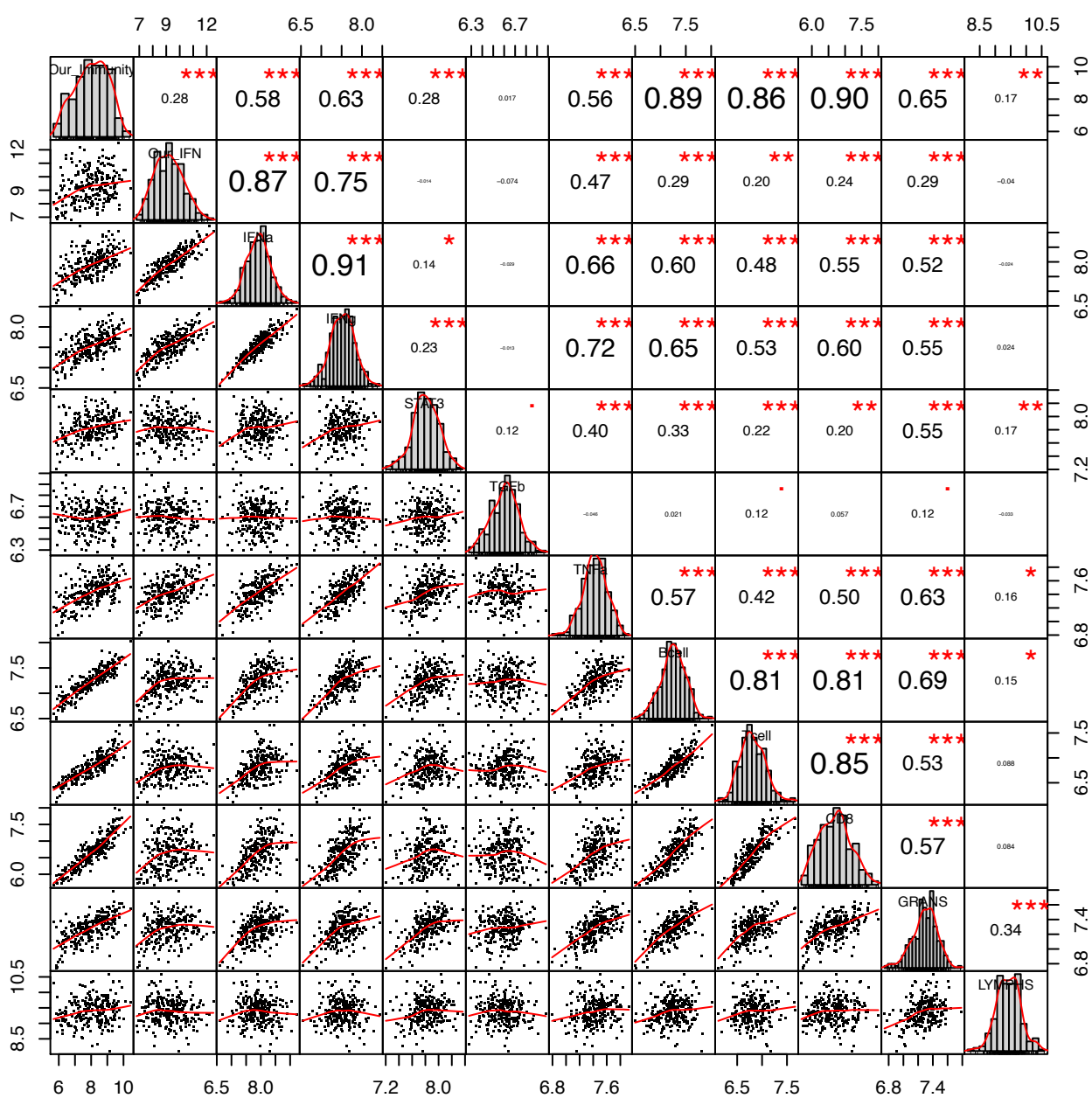

Supplement: S5 Fig — Distribution histograms for our Immune metagenes (Immunity and Interferon) and the immune pathway metagenes published by Gatza et al. (Interferon alpha, Interferon gamma, STAT3, TGF beta, TNF alpha) and Palmer et al. (B Cell, T Cell, CD8 T Cells, Granulocytes, Lymphocytes), Pearson correlation coefficient values and pairwise scatter plots. (PDF) [file pone.0167397.s005.pdf]
