# Supplementary material for: A Stromal Immune Module Correlated with the Response to Neoadjuvant Chemotherapy, Prognosis and Lymphocyte Infiltration in HER2-Positive Breast Carcinoma Is Inversely Correlated with Hormonal Pathways
Source: PLoS One. 2016 Dec 22;11(12):e0167397. doi: 10.1371/journal.pone.0167397 (PMC5178998; doi:10.1371/journal.pone.0167397)
Supplement: S6 Fig — A. Boxplots of gene expression for the Immune metagenes, the immune pathway metagenes (published by Gatza et al. and Palmer et al.) and the PD1, PDL1, CTLA4 metagenes in breast cancer cell lines from the CCLE (A) and the CGP (B). C: Boxplots of the stromal contribution to global gene expression evaluated with PDX RNAseq data (Isella et al.), for each of the gene clusters for our signature. (PDF) [file pone.0167397.s006.pdf]

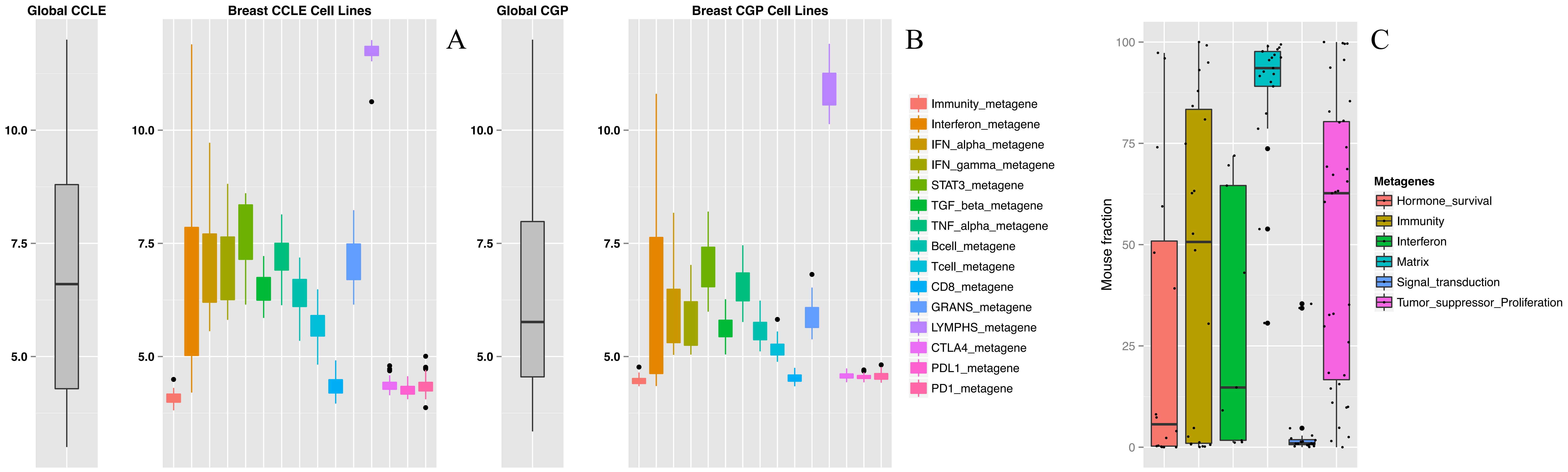
Global CCLE
Breast CCLE Cell Lines
A
Global CGP
Breast CGP Cell Lines
B
C
10.0
7.5
5.0
100
75
50
25
0
Mouse fraction
Immunity_metagene
Interferon_metagene
IFN_alpha_metagene
IFN_gamma_metagene
STAT3_metagene
TGF_beta_metagene
TNF_alpha_metagene
Bcell_metagene
Tcell_metagene
CD8_metagene
GRANS_metagene
LYMPHS_metagene
CTLA4_metagene
PDL1_metagene
PD1_metagene
Metagenes
Hormone_survival
Immunity
Interferon
Matrix
Signal_transduction
Tumor_suppressor_Proliferation
