# Supplementary material for: A Stromal Immune Module Correlated with the Response to Neoadjuvant Chemotherapy, Prognosis and Lymphocyte Infiltration in HER2-Positive Breast Carcinoma Is Inversely Correlated with Hormonal Pathways
Source: PLoS One. 2016 Dec 22;11(12):e0167397. doi: 10.1371/journal.pone.0167397 (PMC5178998; doi:10.1371/journal.pone.0167397)
Supplement: S1 File — (PDF) [file pone.0167397.s007.pdf]

Supporting information file 1

1 Supplementary Methods

1.1 Data

1.1.1 Training and validation sets

We collected 21 publicly available datasets containing raw gene expression data from microarrays (Affymetrix© GeneChip Human Genome HG-U133A and HG-U133Plus2) for 2893 primary human breast cancer samples. The raw data were downloaded from the NCBI Gene Expression Omnibus or ArrayExpress, with the following identifiers: GSE1456 (Pawitan Y.), GSE1561, GSE2034 (Wang Y), GSE2603 (Minn AJ), GSE2990 (Sotiriou C), GSE3494 (Miller LD), GSE5327, GSE5847 (Boersma BJ), GSE7390 (Desmedt C), GSE11121 (Schmidt M), GSE20194, MDA133, GSE2109, GSE7904 (Richardson AL.), GSE12276 (Bos PD), GSE16446 (Juul N), GSE18864 (Juul N), GSE19615 (Juul N), GSE22513 (Bauer A), GSE28796, GSE28821. Raw GE values for each dataset (*n*=21) were normalized independently, for identification of the *HER2*-positive samples in each dataset. The training set included samples hybridized on HGU-133A Affymetrix© arrays (12 datasets, *n*=1921), to eliminate cross-platform discrepancies and to ensure robust normalization. The validation set included samples hybridized on HGU-133Plus2 Affymetrix© arrays (9 datasets, *n*=972).

1.1.2 The Ignatiadis dataset

We collected eight publicly available datasets from the following studies: EORTC10994, I-SPY-1, LBJ/INEN/GEICAM, the MDACC trial, TOP, MAQCII/MDACC, MAQCIII, USO-02103, all of which used Affymetrix GeneChip Human Genome HG-U133A arrays.

Ignatiadis *et al.*[1] used these datasets to analyze the responses of various molecular subtypes of breast cancer to neoadjuvant chemotherapy (anthracycline, with or without taxane; *n*=996). Raw GE values for each dataset were normalized independently, for identification of the *HER2*-positive samples in each dataset. As different sample types were available for gene expression, we chose to keep only tumors sampled by fine needle aspiration, to ensure that the samples studied were homogeneous, as this was the technique used for the majority of tumors in the Ignatiadis dataset (*n*=586).

1.1.3 The METABRIC set

We used the METABRIC — Molecular Taxonomy of Breast Cancer International Consortium — dataset published by Curtis *et al.*[2], which was established for analysis of the prognosis of various molecular subtypes of breast cancer. We normalized the 1992 samples together, using scripts and Rdata provided by the authors. We fitted a linear model (limma R package) to remove the batch effect and probes were filtered according to three criteria: probe quality [3], GC content and presence in more than 5% of the samples.

1.2 Determination of *HER2*-positive status, ER, PR and AR status

The Affymetrix probe 216836_s_at was chosen to provide information about *HER2* expression for the training, validation and Ignatiadis datasets [4]. For the METABRIC dataset, after quality control, the Illumina probe ILMN_2352131 was chosen to provide information about *HER2* expression. We used a threshold value of 1150 for ERBB2 mRNA to identify *ERBB2*-positive patients, as described by Gong[5] for the training and validation datasets, and the bimodal distribution of ERBB2 expression for the Ignatiadis and METABRIC datasets. GE analyses identified 448, 194, 82 and 248 *HER2*-positive samples in the training set, the validation set, the Ignatiadis set and the METABRIC set, respectively. We

identified the ER-, PR- and AR-positive samples in each dataset as follows: the distributions of ER PR and AR expression were analyzed empirically, with a two-component Gaussian mixture model, and parameters for bimodal filtering were estimated with the R Mclust package. A density plot of ER, PR and AR gene expression in each dataset showed a bimodal distribution for ER and PR, but not for AR. The median value was used as the cutoff for AR expression in each dataset.

1.3 Preprocessing of *HER2*-positive samples

1.3.1 Affymetrix© platform

For each dataset (training, validation and prediction), we used the R arrayQualityMetrics package on an Affybatch object to filter out outliers. We excluded samples detected as outliers by at least two of the following methods: distances between arrays, boxplots, relative log expression (RLE), normalized unscaled standard error (NUSE), MA plots, spatial distribution of M. The *HER2*-positive samples were selected and split into training and validation sets. We filtered out 52 outlier samples from the training set and 11 from the validation set. Raw GE values for the *HER2*-positive samples in each dataset (training, validation and prediction) were normalized independently. The optimal microarray probe set to represent a gene was selected with the R JetSet package. This package developed scoring methods for the assessment of each probe set for specificity, coverage, and degradation resistance. For each dataset, batch effects were removed by median centering of each probe set across arrays and the quantile normalization of all arrays separately for each set.

1.3.2 Illumina© platform

We used the Illumina© probe quality score introduced by Barbosa-Morais in 2010 to ensure that all the probes used were of high quality (deleting probes scored as “bad” or “no match”). Probes were filtered on the basis of three criteria: probe quality[3], GC content between 38%

and 64% and presence in more than 5% of the samples (*n*= 20,009 probes). We eliminated any batch effects associated with sample collection sites, by fitting a linear model (R limma package). We then chose the most variable probes as the optimal probe set.

1.4 Statistical analyses

All statistical analyses were performed with R software. Affymetrix© microarrays were normalized with the robust multichip average (RMA) procedure from the EMA R package[6]. Principal component analysis was carried out and heatmaps were generated with the R gplots package.

1.5 Establishment of the *HER2*-positive classifier

1.5.1 Gene selection process

Consensus clustering[7] was applied to the training set, to determine the optimal number of robust gene clusters from the most variable genes (standard deviation >0.8) (ConsensusClusterPlus R package). Cluster robustness was assessed by hierarchical clustering (1,000 iterations) with a ward inner, final linkage and Pearson distance. The optimal number of clusters was determined from the cumulative distribution function (CDF), which plots the corresponding empirical cumulative distribution, defined over the range [0,1], and by calculating the proportional increase in the area under the CDF curve. The number of clusters was set as that at which an increase in cluster number (k) did not lead to a marked increase in CDF area. We calculated Pearson's correlation coefficient for the relationships between genes within the same cluster, to assess the heterogeneity of each gene cluster. The consensus clustering method and hierarchical clustering identified five main gene clusters. Further increases in cluster number yielded no significant increase in the consensus distribution function area. Each gene cluster was tested for gene enrichment (biological process (BP), molecular function (MF), cellular component (CC)) by a conditional test for

overrepresentation, in the R runHyperGO package. The various gene clusters were associated with different gene ontologies). The clusters were named as follows: Immunity cluster (70 genes), Signal/transduction cluster (130 genes), Hormonal/survival cluster (312 genes), Tumor suppressor cluster (65 genes) and Matrix cluster (39 genes). The Matrix and Immunity clusters were the most homogeneous, with strong correlations between the gene expression profiles of most of the genes within each of these clusters (Pearson's correlation coefficients of 0.60 and 0.43, respectively). We then used the String© database (http://string-db.org/help/index.jsp?topic=/org.string-db.docs/ch04.html)[8] to identify biological gene networks. String© is a database of known and predicted protein interactions. The interactions include physical and functional associations derived from four sources: genomic context, high-throughput experiments, conserved coexpression, previous knowledge. For each gene cluster, we excluded genes that were not connected to any of the other genes present in the cluster. We then applied a two-step selection process: 1) we selected strong biological networks, by retaining genes with connection scores of at least 0.7 to each other, according to the String database 2) within each biological network, we then selected groups of genes with correlated patterns of expression, with correlation coefficients of at least 0.5. For this step, we used Cytoscape (http://cytoscapeweb.cytoscape.org), an open-source software platform for visualizing complex networks and integrating them with any type of attribute data. Attribute data, such as correlations, variance and interquartile range, were calculated from the expression data matrix.

After selection, we checked that the various genes selected from the same cluster clustered together again (R package ConsensusClusterPlus). Following biological network-driven gene selection, it became clear that the original Immunity cluster was more accurately described by splitting into two slightly different subclusters (Immunity (*n*=28), Interferon (*n*=11)). This approach yielded an increase in the area under the consensus distribution function (CDF)

curve. The six clusters were renamed as follows, after reclustering: Immunity (n=28), Interferon (n=11), Signal transduction (n=20), Hormonal/survival (n=22), Tumor suppressors/Proliferation (n=36), Matrix (n=21).

1.5.2 Metagene identification

For each dataset (training, validation, Ignatiadis and METABRIC), each gene cluster was used to define a metagene (for the Affymetrix© platform: from the 138 selected genes; for the prognosis set, we used the 136 genes common to the preprocessed prognosis expression matrix and the selected genes matrix from the Affymetrix© platform). Metagene expression was assessed by calculating the median value for the normalized expression values of all probe sets in the respective gene clusters for each sample. For each dataset, we calculated the correlation between expression levels for the various metagenes, using the R psych package. The metagene value for each sample was then classified as corresponding to "high" or "low" expression according to the median value for the metagene.

1.5.3 Classification of HER2-positive samples

In each dataset, hierarchical clustering was applied to the *HER2*-positive GE profiles, using the selected genes to visualize the optimal number of stable *HER2*-positive subtypes (ConsensusClusterPlus R package). We identified five *HER2*-positive subtypes, using the 138-genes signature. We checked the concordance between each of the validation sets and the training set (Pearson's correlation coefficient for the relationship between centroids). Centroid expression values were determined by calculating the mean normalized expression values for all samples in the sample cluster, for each probe set (Figure S2).

1.6 Analysis of the predicted response to chemotherapy prognosis, and the correlation with intratumoral and stromal lymphocyte levels

1.6.1 Analysis of the response to chemotherapy

We assessed the predictive value of our metagenes, by collecting the independent dataset described above (the Ignatiadis set), which contained data for gene expression and clinical variables. We selected *HER2*-positive samples and the data were preprocessed as described above. The following clinical and pathological variables were available and were categorized as follows: age (<50 *versus* ≥50), pre-chemotherapy clinical tumor size (T1 and T2, T3, T4), pre-chemotherapy tumor nodal status (N0 *versus* N1, N2, N3), tumor grade (Grade I and II *versus* III), treatment type, and response to chemotherapy (pathological compete response *versus* no pathological complete response). ER and PR status were assessed by gene expression (as described above). We assessed the expression of the metagenes in each population. Given their unimodal distribution, the expression of each metagene was classified as "low" or "high", based on the median value for the six metagenes. Pathological complete response (pCR) was defined as the disappearance of the invasive component of the primary tumor in one study**[9]** and no residual invasive cancer in the breast and axillary lymph nodes in the other seven studies. Factors predictive of pCR were introduced into a univariate logistic regression model. A multivariate logistic model was then generated. The covariates selected for the multivariate analysis were those with a likelihood ratio test p-value lower than 0.10 in univariate analysis. A backward stepwise selection procedure was used.

We also tested the predictive value of nine immune signatures or metagenes validated as predictive of the response to chemotherapy in *HER2*-positive breast cancer patients: 12 immune-gene signatures [10], Th1 and T-fh metagenes [11], an immune-related 23-gene signature for NAC (IRSN-23) [12], the CXCL13, CCL8 and CXCL9 metagenes [13], the

LCK and IgG combined metagene [14], and the *HER2*-derived prognostic predictor (HDPP)[15]. We performed several multivariate analyses with each signature or metagene independently and the clinical variables significantly associated with pCR in univariate analysis (tumor grade, tumor stage, ER status). We generated a heatmap of the gene expression profiles of each of the above predictive signatures (Figure S4A). The samples were ordered according to our classification of low/high levels of Immunity metagene expression. Expression patterns were similar for the Immunity metagene and for the other predictive gene expression signatures or metagenes, with the exception of Staaf's signature genes.

1.6.2 Prognosis

We assessed the prognostic classification, by collecting the independent dataset described above (METABRIC set), which contained data for gene expression and clinical variables. We selected *HER2*-positive samples and the data were preprocessed as described above. The following clinical and pathological variables were available and were categorized as follows: age (≤45, [45-55], >55), menopausal status, tumor size, tumor grade according to the Elston and Ellis grading system, number of lymph nodes involved (0 (N-, node negative) versus 1 or more lymph nodes involved (N+, node positive)), the Nottingham Prognostic Index score (good prognosis, intermediate prognosis, poor prognosis), treatment type, last follow-up status and the time at which last-follow-up occurred. ER and PR statuses were assessed on the basis of gene expression (as described above). We assessed the expression of the metagenes in each population and the expression of each metagene was classified as "low" or "high", relative to the median value for the six metagenes. Survival analyses were performed by calculating Kaplan-Meier estimates of the survival function. The endpoint of these analyses was breast cancer-specific survival (BCSS) (death from breast cancer). Time-censoring analyses were performed with a right censoring of events from 1 to 20 years. Log-rank tests were used to compare survival curves. Hazard ratios and their associated 95% confidence intervals were

calculated with the Cox proportional hazard model. Variables with a *p*-value for the likelihood ratio test lower than 0.10 in univariate analysis were included in the multivariate model. Backward selection was used to establish the final multivariate model. However, NPI was excluded due to redundancy with the variables size, grade and lymph node status.

Analyses were performed in the whole population, and in the ER-positive, and ER-negative populations. We also assessed the prognostic value of nine immune signatures or metagenes previously validated as predictive of prognosis in *HER2*-positive breast cancer patients: the LCK metagene[14], an immune response module described by Desmedt [16], the T-fh and Th1 metagenes, and CXCL13 alone[11], the stroma-derived prognosis predictor (SDDP)[17]) or signatures developed specifically for *HER2*-positive breast cancers (HDPP [18][18](18)(17)(17)[15], the 105 lymphocyte-associated gene signature [19], the 14-immune gene signature [20]). We generated a heatmap of the gene expression profiles of each of the above prognostic signatures (Figure S4B). The samples were ordered according to our classification of low/high Immunity metagene expression. The expression patterns were similar among signatures, except for the Staaf signature genes, which were associated with poor outcome, and the Finak signature genes associated with a mixed or poor outcome.

1.6.3 Assessment of tumor and stromal infiltrating lymphocytes and Immunity metagene expression in the REMAGUS dataset.

We selected 27 *HER2*-positive breast cancer patients treated by NAC with or without neoadjuvant trastuzumab at our institution during the REMAGUS 02 trial [21]. Core biopsies were obtained before treatment, and separate cores were processed for histology and for RNA extraction, amplification, and hybridization to Affymetrix U133P2 arrays. We selected *HER2*-positive patients on the basis of the expression of the "216836_s_at" probeset. According to the bimodal distribution of ERBB2 expression, 74 of the 226 samples for which

transcriptomic data were available were considered *HER2*-positive. Gene expression data were normalized with the RMA package and batch effects were removed by the median centering of each probe set across arrays and the quantile normalization of all arrays separately for each set. Thirty of these patients were treated at our institution, and pretreatment microbiopsies corresponding to the gene expression chips were retrieved for 27 of them. Histologic microbiopsy specimens were evaluated independently for the presence of a lymphocytic infiltrate by one BCA pathologist (ML) and one physician (ASHP) unaware of the gene expression classification. Intratumoral TILs and stromal TILs were quantified in a semi-quantitative manner as percentages, as previously recommended [22]. Samples were split into Immunity "low" and "high" expression, relative to the median value for the metagene, as described for the other datasets. Intratumoral TIL and stromal TIL percentages were compared between the Immune "high" and Immune "low" subgroups by ANOVA. The correlation between gene expression and intratumoral TIL and stromal TIL percentages was assessed by calculating Pearson's correlation coefficient.

1.6.4 Expression of our gene signature in human breast cancer cell lines

We assessed the expression of our signature in "*in vitro*" models, as a means of validating our classification and its prognostic value. We used the gene expression profiles of the human cancer cell lines from the Cancer Cell Line Encyclopedia (CCLE)[23] of Novartis/the Broad Institute and from the Cancer Genome Project (CGP)[24] of the Sanger Institute. All the cell lines from different tissues were normalized together. The global gene expression signal is shown for breast cancer cell lines from the CCLE (58) and CGP (39). Box plots were generated for the expression of the Immunity and Interferon metagenes, and the metagenes defined by Gatza *et al.* (16) (IFN-alpha, IFN-gamma, STAT3, TGF-beta, TNF-alpha) and Palmer *et al.* (17) (LB, LT, CD8, GRANS, LYMPHS) in the CCLE and CGP.

2 Supplementary results

2.1 Correlations between 138 genes and metagenes

We assessed the correlation of each gene of the 138-gene signature with each of the six metagenes. For the Immunity, Interferon and Matrix metagenes, the expression of several genes was correlated with that of the metagene with correlation coefficients greater than 0.90 (Immunity metagene: CCL5, LCK, CD3D, CD2; Interferon metagene: IFI44L, IFIT1, ISG15, MX1; Matrix metagene: CDH11, FNB1, COL5A1, COL5A2, THBS2). The ESR1 gene was the gene most strongly correlated with the Hormone survival metagene, with a correlation coefficient of 0.77. The ESR1 gene was also inversely correlated with the Immunity metagene, with a correlation coefficient of -0.43.

2.2 Validation of the 138-gene signature

For Affymetrix© arrays, we used the 138 selected genes. For the Illumina© platform, we used 136 common genes. The corresponding heatmaps and the correlation coefficients for the relationships between each sample subgroup centroid in the three validation sets and the corresponding subgroup centroid in the training set are shown in Supplementary Figure 2.

Sample clustering was moderately consistent between the training and validation gene sets. Concordance was high for centroid 1 (high Immunity, low Matrix, low Hormone/survival, concordance from 69 to 79%), but reproducibility was not high for the other centroids in the validation datasets.

2.3 Correlation of Immunity metagene expression with hormonal pathways

The levels of expression of ESR1, PGR and AR as a function of Immunity metagene expression status were compared in the four datasets (see the results section for the training set). The results below are presented with *p*-values for the differences in the Immunity "low"

versus the Immunity “high” group, in the training, validation, Ignatiadis and METABRIC datasets. ESR1 expression was consistently higher in the “Immunity low” subgroup than in the “Immunity high” subgroup ($p< 10^{-16}$, p=0.008, p=0.003, p<0.00001, respectively). PGR expression was also stronger in the Immunity metagene “low” expression group, in three of the four datasets ($p<10^{-6}$, p=0.003, p=0.07 and p=0.02, respectively). Similarly, AR expression was stronger in the Immunity metagene “low” expression group, in two of the four datasets (p=0.0002, p=0.43, p=0.003 and p=0.17, respectively).

We also compared the level of Immunity metagene expression across the four datasets as a function of ER, PR and AR status, as previously described. Immunity metagene expression was significantly stronger in the ER-negative subgroup than in the ER-positive subgroup, in all four datasets ($p<10^{-9}$, p=0.03, p<0.0001 and p=0.02, respectively). PR negativity was associated with higher levels of Immunity metagene expression in all but the Ignatiadis dataset (p=0.0001, p=0.05, p=0.24 and p=0.04, respectively), and AR negativity was associated with stronger Immunity metagene expression in the training and METABRIC datasets ($p=10^{-6}$ and p=0.04, respectively), whereas no such association was observed in the validation and Ignatiadis datasets (p=0.75 and 0.80, respectively).

We also compared the expression levels of each gene of the Immunity metagene separately as a function of ER status. Most of the genes of the Immunity metagene were significantly less strongly expressed in ER-positive than in ER-negative tumors in all four datasets (training set: 27/28 genes, validation set: 19/28, METABRIC: 23/26 genes, and Ignatiadis dataset: 16/28).

We then assessed the proportion of Immunity “low” and Immunity “high” samples across the four datasets as a function of ER status. The samples of ER-positive patients were more likely to be classified as Immunity “low” in all but the validation set (81% *versus* 19%, $p<10^{-9}$; 47%

versus 53%, *p*=0.54; 63% *versus* 37%, *p*<0.001; 61% *versus* 39%, *p*=0.03). Samples from ER-negative patients were more likely to be classified as Immunity "high" in all but the validation set (71% *versus* 29%, $p<10^{-9}$; 48% *versus* 52%, *p*=0.54; 60% versus 40%, *p*<0.001; 63% *versus* 37%, *p*=0.03).

2.4 Prediction of the response to chemotherapy

We also performed similar analyses in the subset of patients that did not receive trastuzumab (*n*=75). The rates of pCR as a function of ER status were 16.3% (7/43) for ER–positive tumors and 40.6% (13/32) for ER-negative tumors (*p*=0.02). After stratification for Immunity metagene status, the pCR rates obtained were significantly different (*p*=0.003): 7.4% (2/27 Immunity low) v 31.3% (5/16 Immunity high) for ER-positive tumors (*p*=0.08) and 16.7% (2/12 Immunity low) versus 55.0% (11/20 Immunity high) for ER-negative tumors (*p*=0.08).

2.5 Correlations between the Immunity metagene and published signatures

String database connections between the Immunity genes and published predictive and prognostic metagenes or immune signatures are provided in Figure S4. The gene intersection was poor, but our immune signature nevertheless appears to be strongly correlated with other published signatures, consistent with the use of similar immune pathways. Pearson's correlation coefficients for the signatures are shown in brackets. The Immunity metagene was strongly correlated with the T-fh metagene (*r*=0.89), the CXCL13 metagene (*r*=0.74), and the LCK metagene (*r*=0.96). In comparisons with prognostic signatures, it was found to be correlated with the Denkert signature (*r*=0.73), the Th1 metagene (*r*=0.75), the IRSN-23 predictor (*r*=0.62), the CXCL9 metagene (*r*=0.70), and the IgG metagene (*r*=0.78), although the coefficients were lower.

References

1. Ignatiadis M, Singhal SK, Desmedt C, Haibe-Kains B, Criscitiello C, Andre F, Loi S, Piccart M, Michiels S, Sotiriou C: Gene modules and response to neoadjuvant chemotherapy in breast cancer subtypes: a pooled analysis. *J Clin Oncol* 2012, 30:1996–2004.

2. Curtis C, Shah SP, Chin S-F, Turashvili G, Rueda OM, Dunning MJ, Speed D, Lynch AG, Samarajiwa S, Yuan Y, Gräf S, Ha G, Haffari G, Bashashati A, Russell R, McKinney S, Langerød A, Green A, Provenzano E, Wishart G, Pinder S, Watson P, Markowetz F, Murphy L, Ellis I, Purushotham A, Børresen-Dale A-L, Brenton JD, Tavaré S, Caldas C, et al.: The genomic and transcriptomic architecture of 2,000 breast tumours reveals novel subgroups. *Nature* 2012, 486:346–352.

3. Barbosa-Morais NL, Dunning MJ, Samarajiwa S a, Darot JFJ, Ritchie ME, Lynch AG, Tavaré S: A re-annotation pipeline for Illumina BeadArrays: improving the interpretation of gene expression data. *Nucleic Acids Res* 2010, 38:e17.

4. Karn T, Metzler D, Ruckhaberle E, Hanker L, Gatje R, Solbach C, Ahr A, Schmidt M, Holtrich U, Kaufmann M, Rody A: Data-driven derivation of cutoffs from a pool of 3,030 Affymetrix arrays to stratify distinct clinical types of breast cancer. *Breast Cancer Res Treat* 2010, 120:567–579.

5. Gong Y, Yan K, Lin F, Anderson K, Sotiriou C, Andre F, Holmes FA, Valero V, Booser D, Pippen JE, Vukelja S, Gomez H, Mejia J, Barajas LJ, Hess KR, Sneige N, Hortobagyi GN, Pusztai L, Symmans WF: Determination of oestrogen-receptor status and ERBB2 status of breast carcinoma: a gene-expression profiling study. *Lancet Oncol* 2007, 8:203–211.

6. Servant N, Gravier E, Gestraud P, Laurent C, Paccard C, Biton A, Brito I, Mandel J, Asselain B, Barillot E, Hupé P: EMA - A R package for Easy Microarray data analysis. *BMC Res Notes* 2010, 3:277.

7. Monti S, et al.: Consensus clustering: a resampling-based method for class discovery and visualization of gene expression microarray data. Mach. *Learn* 2003, 52:91–118.

8. Jensen LJ, Kuhn M, Stark M, Chaffron S, Creevey C, Muller J, Doerks T, Julien P, Roth A, Simonovic M, Bork P, von Mering C: STRING 8--a global view on proteins and their functional interactions in 630 organisms. *Nucleic Acids Res* 2009, 37(Database issue):D412–6.

9. Bonnefoi H, Potti A, Delorenzi M, Mauriac L, Campone M, Tubiana-Hulin M, Petit T, Rouanet P, Jassem J, Blot E, Becette V, Farmer P, André S, Acharya CR, Mukherjee S, Cameron D, Bergh J, Nevins JR, Iggo RD: Validation of gene signatures that predict the response of breast cancer to neoadjuvant chemotherapy: a substudy of the EORTC 10994/BIG 00-01 clinical trial. *Lancet Oncol* 2007, 8:1071–1078.

10. Denkert C, von Minckwitz G, Brase JC, Sinn BV, Gade S, Kronenwett R, Pfitzner BM, Salat C, Loi S, Schmitt WD, Schem C, Fisch K, Darb-Esfahani S, Mehta K, Sotiriou C, Wienert S, Klare P, André F, Klauschen F, Blohmer J-U, Krappmann K, Schmidt M, Tesch H, Kümmel S, Sinn P, Jackisch C, Dietel M, Reimer T, Untch M, Loibl S: Tumor-infiltrating

lymphocytes and response to neoadjuvant chemotherapy with or without Carboplatin in human epidermal growth factor receptor 2-positive and triple-negative primary breast cancers. *J Clin Oncol Off J Am Soc Clin Oncol* 2015, 33:983–991.

11. Gu-Trantien C, Loi S, Garaud S, Equeter C, Libin M, Wind A De, Ravoet M, Buanec H Le, Sibille C, Manfouo-Foutsop G, Veys I, Haibe-Kains B, Singhal SK, Michiels S, Rothé F, Salgado R, Duvillier H, Ignatiadis M, Desmedt C, Bron D, Larsimont D, Piccart M, Sotiriou C, Willard-Gallo K: CD4+ follicular helper T cell infiltration predicts breast cancer survival. *J Clin Invest* 2013, 123:1–20.

12. Sota Y, Naoi Y, Tsunashima R, Kagara N, Shimazu K, Maruyama N, Shimomura A, Shimoda M, Kishi K, Baba Y, Kim SJ, Noguchi S: Construction of novel immune-related signature for prediction of pathological complete response to neoadjuvant chemotherapy in human breast cancer. *Ann Oncol Off J Eur Soc Med Oncol ESMO* 2014, 25:100–106.

13. Stoll G, Enot D, Mlecnik B, Galon J, Zitvogel L, Kroemer G: Immune-related gene signatures predict the outcome of neoadjuvant chemotherapy. *Oncoimmunology* 2014, 3:e27884.

14. Rody A, Karn T, Liedtke C, Pusztai L, Ruckhaeberle E, Hanker L, Gaetje R, Solbach C, Ahr A, Metzler D, Schmidt M, Müller V, Holtrich U, Kaufmann M: A clinically relevant gene signature in triple negative and basal-like breast cancer. *Breast Cancer Res BCR* 2011, 13:R97.

15. Staaf J, Ringnér M, Vallon-Christersson J, Jönsson G, Bendahl P-O, Holm K, Arason A, Gunnarsson H, Hegardt C, Agnarsson BA, Luts L, Grabau D, Fernö M, Malmström P-O, Johannsson OT, Loman N, Barkardottir RB, Borg A: Identification of subtypes in human epidermal growth factor receptor 2--positive breast cancer reveals a gene signature prognostic of outcome. *J Clin Oncol Off J Am Soc Clin Oncol* 2010, 28:1813–1820.

16. Desmedt C, Haibe-Kains B, Wirapati P, Buyse M, Larsimont D, Bontempi G, Delorenzi M, Piccart M, Sotiriou C: Biological processes associated with breast cancer clinical outcome depend on the molecular subtypes. *Clin Cancer Res* 2008, 14:5158–5165.

17. Finak G, Bertos N, Pepin F, Sadekova S, Souleimanova M, Zhao H, Chen H, Omeroglu G, Meterissian S, Omeroglu A, Hallett M, Park M: Stromal gene expression predicts clinical outcome in breast cancer. *Nat Med* 2008, 14:518–527.

18. Teschendorff AE, Miremadi A, Pinder SE, Ellis IO, Caldas C: An immune response gene expression module identifies a good prognosis subtype in estrogen receptor negative breast cancer. *Genome Biol* 2007, 8:R157.

19. Alexe G, Dalgin GS, Scanfeld D, Tamayo P, Mesirov JP, DeLisi C, Harris L, Barnard N, Martel M, Levine AJ, Ganesan S, Bhanot G: High expression of lymphocyte-associated genes in node-negative HER2+ breast cancers correlates with lower recurrence rates. *Cancer Res* 2007, 67:10669–10676.

20. Perez EA, Thompson EA, Ballman KV, Anderson SK, Asmann YW, Kalari KR, Eckel-Passow JE, Dueck AC, Tenner KS, Jen J, Fan J-B, Geiger XJ, McCullough AE, Chen B, Jenkins RB, Sledge GW, Winer EP, Gralow JR, Reinholz MM: Genomic Analysis Reveals That Immune Function Genes Are Strongly Linked to Clinical Outcome in the North Central

Cancer Treatment Group N9831 Adjuvant Trastuzumab Trial. *J Clin Oncol Off J Am Soc Clin Oncol* 2015.

21. De Cremoux P, Valet F, Gentien D, Lehmann-Che J, Scott V, Tran-Perennou C, Barbaroux C, Servant N, Vacher S, Sigal-Zafrani B, Mathieu M-C, Bertheau P, Guinebretière J-M, Asselain B, Marty M, Spyratos F: Importance of pre-analytical steps for transcriptome and RT-qPCR analyses in the context of the phase II randomised multicentre trial REMAGUS02 of neoadjuvant chemotherapy in breast cancer patients. *BMC Cancer* 2011, 11:215.

22. Salgado R, Denkert C, Demaria S, Sirtaine N, Klauschen F, Pruneri G, Wienert S, Van den Eynden G, Baehner FL, Penault-Llorca F, Perez EA, Thompson EA, Symmans WF, Richardson AL, Brock J, Criscitiello C, Bailey H, Ignatiadis M, Floris G, Sparano J, Kos Z, Nielsen T, Rimm DL, Allison KH, Reis-Filho JS, Loibl S, Sotiriou C, Viale G, Badve S, Adams S, et al.: The evaluation of tumor-infiltrating lymphocytes (TILs) in breast cancer: recommendations by an International TILs Working Group 2014. *Ann Oncol Off J Eur Soc Med Oncol ESMO* 2015, 26:259–271.

23. Barretina J, Caponigro G, Stransky N, Venkatesan K, Margolin AA, Kim S, Wilson CJ, Lehár J, Gregory V, Sonkin D, Reddy A, Liu M, Murray L, Michael F, Monahan JE, Morais P, Meltzer J, Korejwa A, Jané- J, Mapa FA, Thibault J, Bric-furlong E, Raman P, Engels IH, Cheng J, Yu GK, Yu J, Jr PA, Silva M De, Jagtap K, et al.: NIH Public Access of anticancer drug sensitivity. 2012, 483:603–607.

24. Garnett MJ, Edelman EJ, Heidorn SJ, Greenman CD, Dastur A, Lau KW, Greninger P, Thompson IR, Luo X, Liu Q, Iorio F, Surdez D, Chen L, Milano RJ, Bignell GR, Tam AT, Davies H, Stevenson J a, Barthorpe S, Lutz SR, Kogera F, Lawrence K, Mclaren-douglas A, Mironenko T, Thi H, Richardson L, Zhou W, Hur W, Yang W, Deng X, et al.: Europe PMC Funders Group Systematic identification of genomic markers of drug sensitivity in cancer cells. *Nature* 2012, 483:570–575.
